# MicroRNA Profile of HCV Spontaneous Clarified Individuals, Denotes Previous HCV Infection

**DOI:** 10.3390/jcm8060849

**Published:** 2019-06-14

**Authors:** Óscar Brochado-Kith, Alicia Gómez Sanz, Luis Miguel Real, Javier Crespo García, Pablo Ryan Murúa, Juan Macías, Joaquín Cabezas González, Jesús Troya, Juan Antonio Pineda, María Teresa Arias Loste, Victorino Díez Viñas, María Ángeles Jiménez-Sousa, Luz María Medrano de Dios, Isabel Cuesta De la Plaza, Sara Monzón Fernández, Salvador Resino García, Amanda Fernández-Rodríguez

**Affiliations:** 1 Unit of Viral Infection and Immunity, National Center for Microbiology, Institute of Health Carlos III, Majadahonda, 28220 Madrid, Spain; obrochado@isciii.es (Ó.B.-K.); agsmm73@hotmail.com (A.G.S.); jimenezsousa@isciii.es (M.Á.J.-S.); luzmedranodios@gmail.com (L.M.M.d.D.); 2 Unidad Clínica de Enfermedades Infecciosas, Hospital Universitario de Valme, 41014 Sevilla, Spain; lmreal67b@gmail.com (L.M.R.); juan.macias.sanchez@gmail.com (J.M.); japineda@telefonica.net (J.A.P.); 3 Gastroenterology and Hepatology Department, Hospital Universitario Marques de Valdecilla, 39008 Santander, Spain; javiercrespo1991@gmail.com (J.C.G.); joaquin.cabezas@scsalud.es (J.C.G.); mteresa.arias@scsalud.es (M.T.A.L.); 4 Institute Valdecilla (IDIVAL), School of Medicine, University of Cantabria, 39005 Santander, Spain; 5 Internal Medicine Service, University Hospital Infanta Leonor, School of Medicine, Complutense University of Madrid, Gregorio Marañón Health Research Institute, 28009 Madrid, Spain; pabloryan@gmail.com (P.R.M.); jestrogar@hotmail.com (J.T.); victorino.diez@gmail.com (V.D.V.); 6 Bioinformatics Unit, Unidades Comunes Científico Técnicas, Institute of Health Carlos III, Majadahonda, 28220 Madrid, Spain; isabel.cuesta@isciii.es (I.C.D.l.P.); smonzon@isciii.es (S.M.F.)

**Keywords:** chronic hepatitis C, spontaneous HCV clarification, MicroRNAs, lipid metabolism

## Abstract

Factors involved in the spontaneous cleareance of a hepatitis C (HCV) infection are related to both HCV and the interaction with the host immune system, but little is known about the consequences after a spontaneous resolution. The main HCV extrahepatic reservoir is the peripheral blood mononuclear cells (PBMCs), and their transcriptional profile provides us information of innate and adaptive immune responses against an HCV infection. MicroRNAs regulate the innate and adaptive immune responses, and they are actively involved in the HCV cycle. High Throughput sequencing was used to analyze the miRNA profiles from PBMCs of HCV chronic naïve patients (CHC), individuals that spontaneously clarified HCV (SC), and healthy controls (HC). We did not find any differentially expressed miRNAs between SC and CHC. However, both groups showed similar expression differences (21 miRNAs) with respect to HC. This miRNA signature correctly classifies HCV-exposed (CHC and SC) vs. HC, with the has-miR-21-3p showing the best performance. The potentially targeted molecular pathways by these 21 miRNAs mainly belong to fatty acids pathways, although hippo signaling, extracellular matrix (ECM) interaction, proteoglycans-related, and steroid biosynthesis pathways were also altered. These miRNAs target host genes involved in an HCV infection. Thus, an HCV infection promotes molecular alterations in PBMCs that can be detected after an HCV spontaneous resolution, and the 21-miRNA signature is able to identify HCV-exposed patients (either CHC or SC).

## 1. Introduction

Nearly 71 million people live with chronic hepatitis C (CHC) infections, and 1.75 million new hepatitis C virus (HCV) infections emerged worldwide [1]. Around 35% of patients with an acute HCV infection will spontaneously clear the virus within six months of infection without any treatment [1,2]. Clinical features for this viral clarification are related to HCV interactions with the host innate/adaptive immune system and host genetic factors. Thus, a low clearance rate is associated with the male gender, HIV coinfection, hepatitis B virus (HBV) coinfection, specific genetic background as Afro-American ethnicity, non-genotype 1 infection, older age, and high alcohol or drug consumption [3].

Nonetheless, little is known about the cost for the immune system after a clarification of an HCV infection. Regarding cellular response, CD4+ and CD8+ T lymphocyte reactivity to HCV infections persist for decades, although humoral responses seem to gradually decrease and disappear after recovery from an HCV infection [4]. However, few studies have addressed these issues and their clinical consequences with discrepant results. Patients cured of hepatitis C through treatment showed a higher mortality rate overall than the general population [5,6], while mortality in a Danish cohort with cleared HCV infections was similar to the general population [6]. Also, patients with a chronic HCV infection are at higher risk of mortality and liver-related death than those patients who cleared the infection [7].

Genetic background plays a key role in the antiviral immune response against HCV infection, and epigenetic factors such as microRNAs (miRNAs) expression are essential [8]. These small RNAs regulate the innate and adaptive immune response, and host miRNAs can directly target viral genomes or cellular factors to positively or negatively regulate viral infection [9]. HCV relies heavily on cellular factors for its life cycle, such as some host miRNAs which directly target its genome to enhance viral replication and translation [10]. Also, an HCV infection directly impacts on cellular miRNAs expression, disrupting innate immunity pathways to create a permissive environment for its viral replication. Thus, some cellular miRNAs that repress essential HCV cofactors are downregulated by HCV infection [8].

Although HCV mainly replicates within the liver, an extrahepatic replication of HCV has been described in vitro and in vivo in a variety of cells, including peripheral blood mononuclear cells (PBMCs), where HCV-RNA has been detected [11,12,13]. As PBMCs are one of the main HCV extrahepatic reservoirs, the HCV lymphotropism leads to abnormal B-cell activation and the development of lymphoproliferative and autoimmune disorders [14], such as non-Hodgkin’s lymphoma and mixed cryoglobulinemia, which is the most common extrahepatic manifestation of a chronic HCV infection [15]. The HCV lymphotropic variants likely constitute a distinct but parallel source for viral persistence and immune escape within chronically infected patients [12]. Also, the dysfunction of HCV-infected PBMCs leads to immune function decline, and it becomes more difficult for the host to clear intrahepatic HCV [16]. Moreover, recent studies have shown that PBMCs may reflect the alterations of intracellular pathways occurring during HCV-related liver diseases [17], being the innate antiviral response against acute HCV not restricted to the liver [18].

Therefore, we propose that the miRNA profile of PBMC from HCV-exposed patients could give us insight into deregulated miRNAs after HCV resolution and their possible immune consequences. Thus, we aim to analyze the expression profile of miRNAs from HCV chronic naïve patients and individuals who spontaneously resolved an HCV infection with respect to healthy donors. The analysis of differentially expressed miRNAs in participants with different HCV history allowed us to identify a common HCV signature in chronic and spontaneous clearance patients and, therefore, their putative deregulated pathways.

## 2. Experimental Section

An extensive description of the material and methods can be found in the Supplementary Materials.

Samples were recruited from Hospital Universitario Virgen de Valme (Seville), Hospital Universitario Marqués de Valdecilla (Santander), and Hospital Universitario Infanta Leonor (Madrid) from 2014 to 2017. All samples were processed at the National Center for Microbiology (Majadahonda). The study protocol conformed to the ethical guidelines of the 1975 Declaration of Helsinki as reflected in a priori approval by the Ethics Committee of Institute of Health Carlos III, number CEI PI 11_2015-V4, approved on 2 September 2015. Written informed consent was obtained from all patients involved.

### 2.1. Patient Groups

Caucasian patients (*n* = 96) were recruited and grouped by (a) HCV spontaneous clarifiers (SC), individuals who spontaneously resolved an HCV infection (positive serum antibody and negative PCR), with a minimum of six months of follow-up from the diagnosis, and remaining as such thereafter; (b) chronic hepatitis C (CHC) treatment-naïve patients (detectable HCV RNA by PCR for at least six months); and (c) healthy controls (HC) that were never infected with HCV (antibody and PCR negative). All groups were gender balanced to avoid sex bias, and controls were age-matched with SC and CHC groups. Only participants with no advanced liver fibrosis were selected to have a homogeneous cohort and to limit confounding factors.

The general exclusion criteria for all group of patients are as follows: individuals below 18 years old; previously HCV treatment; clinical evidence of hepatic decompensation; alcohol-induced liver injury; HBV-associated antigen/antibody or anti-HIV antibody; active drug or alcohol addiction; opportunistic infections; and other concomitant diseases such as diabetes, nephropathies, autoimmune disease, hemochromatosis, cryoglobulinemia, primary biliary cirrhosis, Wilson´s disease, α-antitrypsin deficiency, neoplasia, and pregnancy.

### 2.2. Clinical Records

HCV-related clinical and epidemiological data were obtained from medical records as the year of infection, time since spontaneous clarification, route of transmission, fibrosis stage, HCV viral load, HCV genotype, and genotype of rs12979860 polymorphism at *interferon lambda 4 (gene/pseudogene)* (*IFNL4*). The liver stiffness measurement (LSM) was assessed by transient elastometry (FibroScan^®^, Echosens, Paris, France) and expressed in kilopascals (kPa). Subjects were stratified according to cut-offs of LSM: <7.1 kPa (F0–F1: absence or mild fibrosis) and 7.1–9.4 kPa (F2: significant fibrosis). The clinical characteristics of metabolic status and biochemical parameter of liver function were also recorded (see Clinical Records at Supplementary Material).

### 2.3. Collection of Biological Samples

PBMCs were isolated within the first 4 h after blood extraction. The total RNA, including miRNAs, was isolated with the miRNeasy Mini kit (Qiagen). Quality and integrity were evaluated by the Bioanalyzer 2100 with Agilent RNA 6000 Nano kit (Agilent).

### 2.4. Analysis of miRNA Expression by High Throughput Sequencing

Small RNA library synthesis and sequencing were performed at the Centre for Genomic Regulation (CRG) at Barcelona (Spain). Briefly, small RNA libraries were constructed with Illumina’s TruSeq Small RNA kit v.4 (Illumina) from 1 microgram of total RNA, following manufacturer´s instructions. Sequencing was performed on the Illumina HiSeq2500, Single-Read, 50 nts (1 × 50). Four pools of 24 barcoded samples were prepared, and each pool was sequenced on one line of the same run to limit batch effect.

Raw data were analyzed with a specific bioinformatic pipeline for the identification of known and novel miRNAs, which is extensively described in the Supplementary Material. Quality control was performed with FastQC (v.0.11.3); adapter sequences were trimmed with cutadapt (v.1.13) and processed with miRDeep2 (v.0.0.7), which identify known and novel miRNAs. miRBase v.20 and GRCh38 reference genome assembly were used as reference databases.

### 2.5. miRNA Validation by qRT-PCR

Sixty-six additional individuals were selected for validation purpose, where 22 individuals corresponded to each group of study (HC, SC, and CHC). Five hundred ng of total RNA was reverse transcribed into complementary DNA with the qScript microRNA cDNA synthesis kit, following the manufacturer instructions. Individual miRNAs were quantified in a SYBR green quantitative PCR. Sequences of the miRNA nucleotides were extracted from the miRBase Release 21 (www.mirbase.org) (Table S6). The PCR efficiency of each miRNA amplicon was evaluated, and efficiencies higher than 1.9 were accepted. Real time reactions were performed on a Roche LightCycler 480. Endogenous control selection was performed according to the stability of their gene expression in the three groups of study. Raw data were exported to Factor qPCR to normalize and remove multiplicative between-run variations of PCR experiments with multiple plates. [19]. Data were analyzed using the ΔΔCT method. For extended information, see the Experimental Section in the Supplementary Materials.

### 2.6. miRNA-Based Target Prediction and Pathway Enrichment Analysis

The web-based computational tool DIANA-miRPath v3.0 was used for the *in silico* target identification of the significantly differentially expressed (SDE) miRNAs, which is based on experimentally supported target predictions. This tool also performs a pathway union analysis of miRNAs targets by the Kyoto Encyclopedia of Genes and Genomes (KEGG) pathways. Enrichment p-values (Fischer’s exact test with hypergeometric distribution) were corrected for the false discovery rate (FDR) (*p* ≤ 0.05). Next, the SDE miRNAs were subjected to a target-based pathway enrichment analysis to identify miRNA-mRNA regulatory networks with miRNet.

### 2.7. Statistical Analyses

Thirty-two samples for each group (HC, SC, and CHC) were sequenced. The sample size for each group was calculated according to the RnaSeqSampleSize calculator [20], which established a minimum of 27 samples per group. Calculates were performed by using the following parameters: 100 minimum average read counts; an estimated dispersion of 0.4, which is used for human data; and a minimum fold change of 2. Also, a specific analysis on miRNA sequencing shows that 32 individuals per group are more than enough to detect a minimum 2 fold change with a false discovery rate of 5% [21].

We used Principal Component Analysis (PCA) to visualize whether the experimental samples were clustered according to the groups of patients and to identify the unwanted source of noise.

Currently, there are no specific software packages designed to normalize miRNA sequencing data; for this reason, three normalization methods commonly used for RNA sequencing analysis have been used: (1) reads per kilobase million (RPKM), by the Differential gene expression analysis based on the negative binomial distribution (DESeq) R package (v.1.28.0); (2) trimmed mean of M-values normalization method (TMM), by the Empirical Analysis of Digital Gene Expression Data in R (EdgeR) (v.3.18.1); and (3) upper quantile normalization (UPERQ), by NOISeq R package (v.2.14.1).

SDE miRNAs were calculated by a fold change (FC) > 2 and a statistically significant *t*-test *p* value < 0.05 adjusted by FDR using the Benjamin–Hochberg correction. (1) DESeq fit a generalized linear model for each miRNA with the fold change estimate shrunken by empirical Bayes; the function *nbionmTest* was used to estimate differences between groups. (2) edgeR used a likelihood ratio test for determining the differential expression among groups of patients, and it was performed with the *glmFit()* and *glmLRT()* functions. (3) NOISeqBIO is a nonparametric method that improves the control of the high FDR in experiments with biological replicates.

A Venn Diagram was performed to determine the overlapping of SDE miRNAs, which were defined as common SDE miRNAs in the three methods of normalization and statistical analysis. The overlapping list of each group of comparison was selected for a subsequent analysis.

We also conducted a partial least square discriminant analysis (PLS-DA), which is used for predictive and descriptive modeling as well as for discriminative variable selection [22] in high-dimensional data. PLS-DA is a supervised learning method which uses the normalized miRNA expression profile for analysis. We used the HCV-exposed (SC and CHC) or non HCV-exposed group as the response variable and the expression profile of the SDE miRNAs between those groups as explanatory variables. Also, the Variable Importance for the Projection (VIP) criterion was used to identify the contribution of each specific predictor for both the explained variability on the response and the explained variability on the predictors. PLS-DA and VIP were performed with the caret package (v.6.0-81) included in the R Project for Statistical Computing.

For the descriptive study of clinical and demographic data of the patients, continuous variables were summarized as the median and the interquartile range and categorical variables were summarized as absolute numbers (%). Significant differences between categorical data were calculated using the chi-squared test or Fisher´s exact test. The Kruskal–Wallis and Mann–Whitney U tests were used to compare continuous variables among independent groups. IBM^®^ SPSS Statistics (v.22) and statistical software R (v.3.2.0) (www.r-project.org) were used for all statistical analyses.

## 3. Results

### 3.1. Clinical Characteristics of Each Group of Patients

Table 1 shows the HCV-related epidemiological and clinical characteristics of the three groups of participants included in our study: CHC, SC, and HC. The time since clearance of SC individuals ranged from 0.6 to 47 years. Regarding CHC patients, the time of infection ranged from 1 to 38 years. Only some variables showed significant differences between groups (*p* < 0.05), such as the transmission route of HCV infection, where CHC showed a higher proportion of IDUs. Moreover, the SC group showed a significantly higher proportion of the favorable *IFNL4* rs12979860 CC genotype than CHC and HC, as expected.

Table 2 shows the metabolic status of the patients. Most of the data were only available for CHC and SC patients. The lipid profile analysis did not show significant differences between groups. High AIP values were found in both groups. Biochemical parameters related to liver function such as liver enzymes GOT, GPT, GPT > 40 mg/dL, GGT, and APRI index, showed higher values in the CHC group (*p* = 0.002, *p* < 0.001, *p* = 0.005, *p* = 0.039, and *p* = 0.032, respectively).

### 3.2. Differentially Expressed miRNAs Between Groups

The raw sequencing data have been deposited in the ArrayExpress repository (EMBL-EBI) under accession number E-MTAB-8023. We identified a total of 1881 known miRNAs (of 2822 recorded in miRBase v.20) with more than 5 counts, plus 137 putative de novo miRNAs that fulfilled the selection criteria (see details in the Supplementary Material). The 137 de novo miRNAs are listed in Table S1. On average, 10.9 million reads per sample were obtained, which is an appropriate depth for an expression analysis [23]. The 54% of the reads could be mapped to the reference genome, (quantifier log), and 66.6% of the miRNAs in miRBase were detected. Also, we have performed our analysis on a large data set (96 samples). The number of samples plays a more significant role in determining the power than the read counts [24], as increasing the number of replicates significantly improves the detection power over an increased sequencing depth.

**Figure 1:**
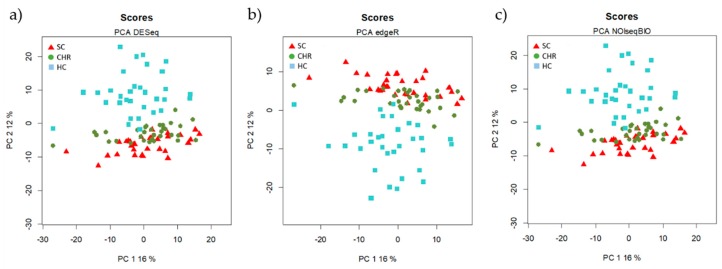
Unsupervised Principal Component Analysis (PCA) of the data normalized by the three methods of normalization (**a**) DESeq, (**b**) edgeR, and (**c**) NOIseq. Each symbol represents the miRNA profile from one of the 96 participants of the study. All methods show an overlap between HCV chronic naïve (CHC) patients and spontaneously clarified HCV (SC) individuals.

The principal component analysis (PCA) of each normalized set of data (by DESeq, edgeR, and NOISseq) was performed (Figure 1). In the three PCAs, the HC group formed independent clusters with the three normalization methods and both CHC and SC grouped together.

Differential expressions analyses’ results showed similar fold changes for each normalization and statistical method (Table S2).

#### 3.2.1. miRNAs Associated with HCV Spontaneous Clarification

**Figure 2:**
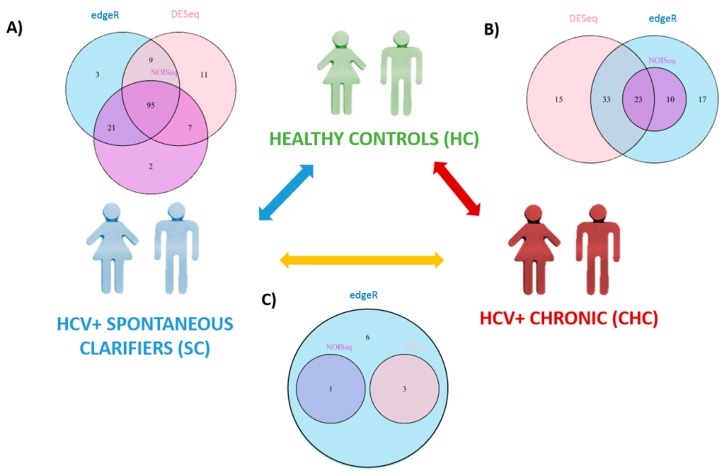
Statistically significant differentially expressed (SDE) miRNAs calculated by different methods of analysis—DESeq circles in pink, edgeR circles in blue, and NOISeq circles in purple—between each group of study: HC, SC, and CHC. Venn Diagrams show common SDE miRNAs in each analysis. (**A**) SDE miRNAs between HC and SC; (**B**) SDE miRNAs between HC and CHC patients; and (**C**) SDE miRNAs between SC and CHC patients.

**Figure 3:**
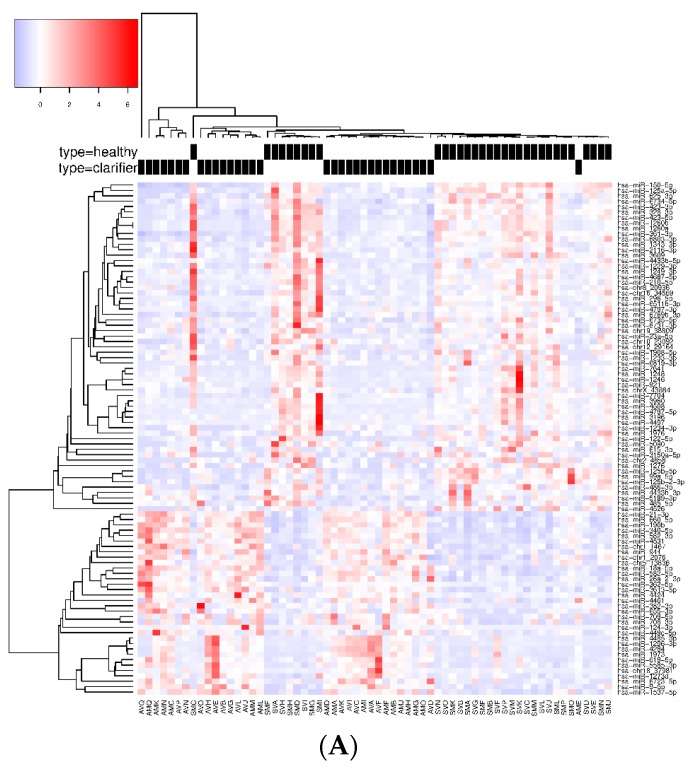

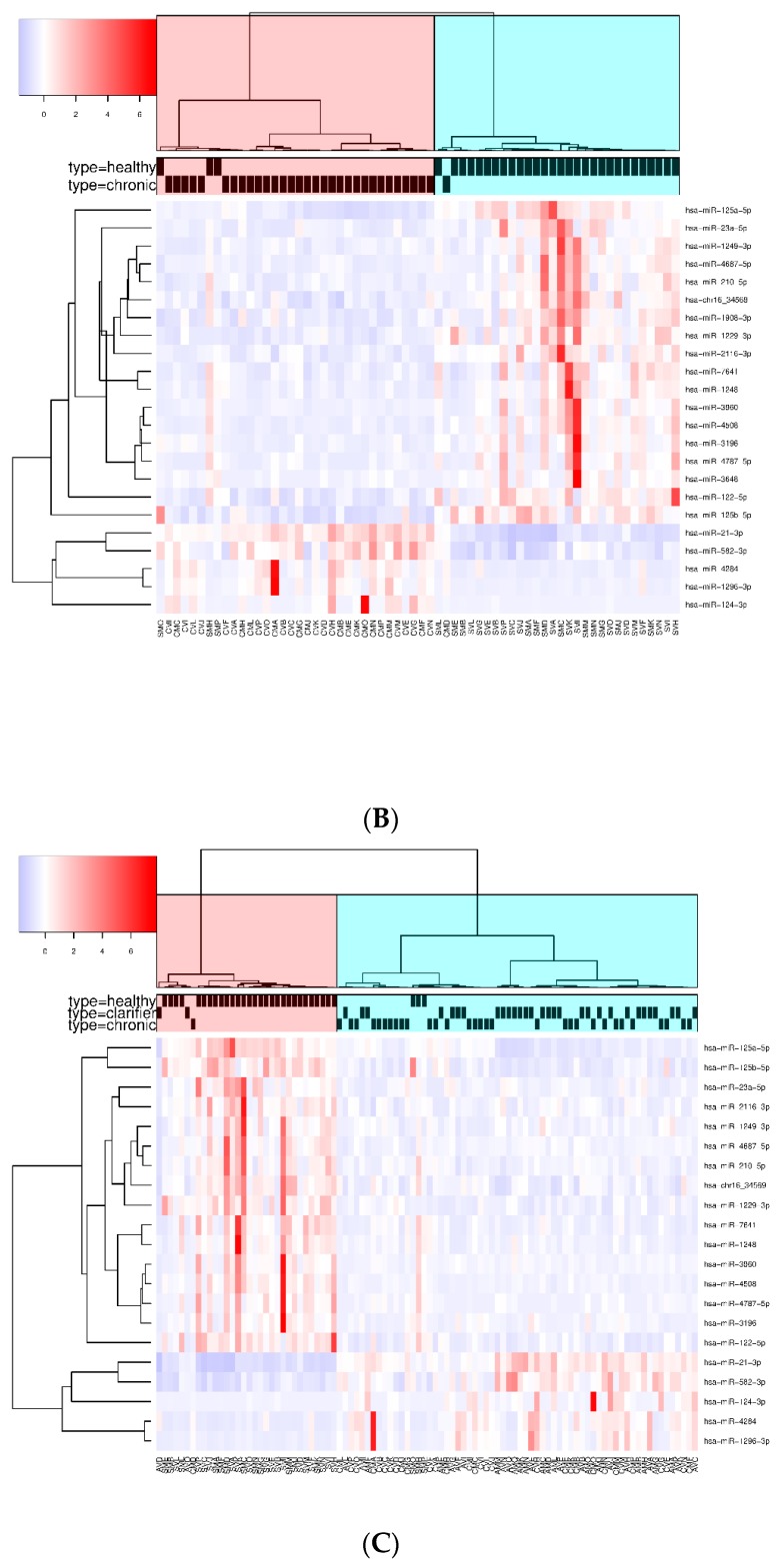
Heatmap and unsupervised clustering of SDE miRNAs compared between groups of study. Only miRNAs common to the three methods of analysis are shown. Columns represent each sample, while rows correspond to SDE miRNAs between groups of comparison. The miRNA clustering tree is shown on the left, and the sample clustering tree is shown at the top. The color scale at the top left illustrates the relative expression level of SDE miRNAs, with red indicating a higher expression level and blue a lower expression level. (**A**) HC versus SC; (**B**) HC vs. CHC patients; and (**C**) SDE common to SC vs. HC and CHC vs. HC.

When we compared the SC vs. HC groups, 95 SDE miRNAs were common to the three analysis, where 122 miRNAs were identified by DESeq, 128 miRNAs by edgeR, and 125 miRNAs by NOISeq (Figure 2A). The identification of all SDE miRNAs and their fold changes are listed in Supplementary Table S3. The hierarchical cluster analysis of the common 95 SDE miRNAs showed distinct patterns of miRNA expression between groups of comparison. We identified 61 miRNAs downregulated and 34 upregulated in SC (Figure 3A). From the 95 SDE miRNAs, 11 of them were de novo miRNAs (hsa-chr1_2076, hsa-chr1_1467, hsa-chr2_4858, hsa-chr5_13836, hsa-chr8_20936, hsa-chr10_23092, hsa-chr12_29164, hsa-chr16_34569, hsa-chr18_37981, hsa-chr19_38809, and hsa-chrX_43884), located at chromosomes 1, 2, 5, 8, 10, 12, 16, 18, 19, and X, respectively.

#### 3.2.2. miRNAs Associated with HCV Chronic Infection

When CHC and HC were compared, 71 SDE miRNAs were identified by DESeq, 83 by edgeR, and 33 by NOISeq. The three methods shared 23 SDE miRNAs (Figure 2B). The hierarchical cluster analysis of the common 23 SDE miRNAs showed two main clusters which corresponded to the patient groups (HC and CHC) (Figure 3B). The majority of SDE miRNAs (*n* = 18) were downregulated in CHC compared to HC, and only five miRNAs were upregulated during an active HCV infection (Supplementary Table S4). One of the downregulated SDE miRNAs corresponded to one putative de novo miRNA located at chromosome 16 (hsa-chr16_34569). Twenty-one out of 23 SDE miRNAs were the same to those identified in the SC comparison with respect to HC.

#### 3.2.3. Analysis of miRNAs Between CHC and SC Patients

Similar miRNA expression patterns were identified in SC subjects and CHC patients, as the differential expression analysis did not find any common SDE miRNAs between the three statistical methods (Figure 2C). EdgeR identified ten SDE miRNAs, DESeq three, and NOISeq one. Three SDE miRNAs were common to DESeq and edgeR, and only one was common to NOISeq and edgeR (Figure 2C, Supplementary Table S5). These data substantiated our PCA findings and were as commented above: SC and CHC clustered together and separated from HC. Therefore, our data suggest that HCV-exposed patients (SC and CHC) show a specific miRNA profile.

#### 3.2.4. Common miRNAs in CHC and SC versus HC Subjects

**Figure 4:**
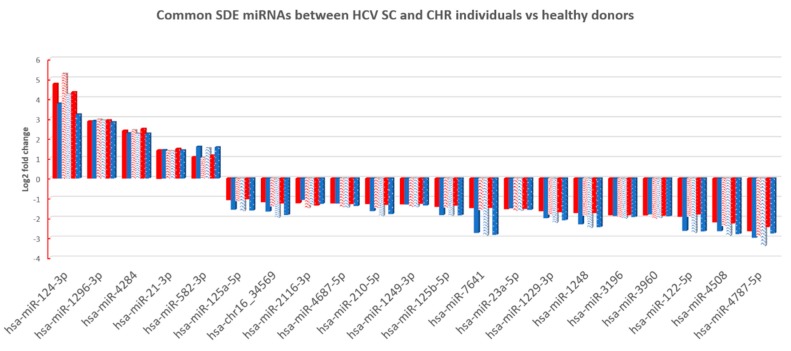
The log2 fold change (FC) of SDE 21 miRNAs between CHC patients (columns in red) and SC individual’s (columns in blue) vs. HC for each method of analysis (columns with different patterns). Similar log2FC were identified in all cases.

Finally, we searched for miRNA patterns shared by HCV-exposed (SC and CHC) individuals. To achieve this goal, we compared the common SDE miRNAs identified between SC and HC (95 SDE miRNAs), with the common SDE miRNAs between CHC and HC (23 SDE miRNAs). As a result, 21 SDE miRNAs were identified as mutual to both analyses, indicating an HCV-specific signature of miRNAs in PBMCs. From the 21 miRNAs, 16 were downregulated and five were upregulated in HCV+ exposed (CHC and SC groups). Also, the heatmap and unsupervised hierarchical clustering of these 21 common SDE miRNAs showed us that CHC patients and SC individuals clustered together and separated from HC (Figure 3C). Therefore, almost all dysregulated miRNAs detected in CHC (except hsa-miR-3648 and hsa-miR-1908-3p) were also disrupted in SC individuals, suggesting a similar miRNA-mediated posttranscriptional regulation in individuals who have been exposed to an HCV+ infection, either to an acute or chronic active infection. Additionally, the fold changes detected for the 21 SDE miRNAs were highly similar in both comparisons, indicating an analogous dysregulation of miRNA-related pathways (Figure 4). Only hsa-miR-7641 showed slight differences in the magnitude of log2FC, not in the direction, but these differences were not statistically significant.

**Figure 5:**
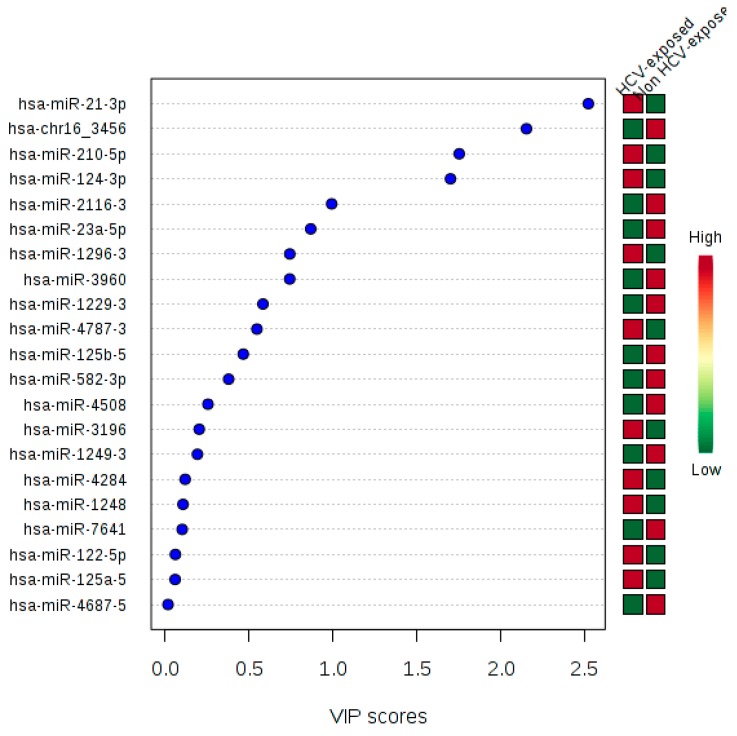
Variable Importance in Projection (VIP) calculated for each 21 SDE miRNAs.

To explore the discrimination ability of these 21 miRNAs, we performed a PLS-DA analysis. The classification between HCV-exposed (SC and CHC) and non-exposed (HC) groups were done without no error, with an accuracy = 1, sensitivity = 1, and specificity = 1 (*p* < 0.001). The VIP values for each miRNA is shown in Figure 5. The hsa-miR-21-3p showed the highest VIP score. Therefore, this 21 SDE miRNAs profile is adequate for discrimination between previously HCV-exposed and non-exposed individuals.

### 3.3. miRNA Validation

Two miRNAs were selected for validation in a new data set, the has-miR-21-3p, which is the miRNA with the highest VIP score, and the has-miR-23a-5p. The endogenous control gene with the best stability value was the RNU44. Patients characteristics can be accessed in Supplementary Table S7. Both miRNAs were validated, showing a reduced expression in HC vs. HCV-exposed (SC and CHC) individuals and no statistically significant differences between CHC and SC (see Supplementary Materials, Figure S1 and Table S8).

### 3.4. Functional Enrichment Analysis of Targets from miRNAs Associated with HCV Spontaneous Clarification and Chronic HCV Infection

**Figure 6:**
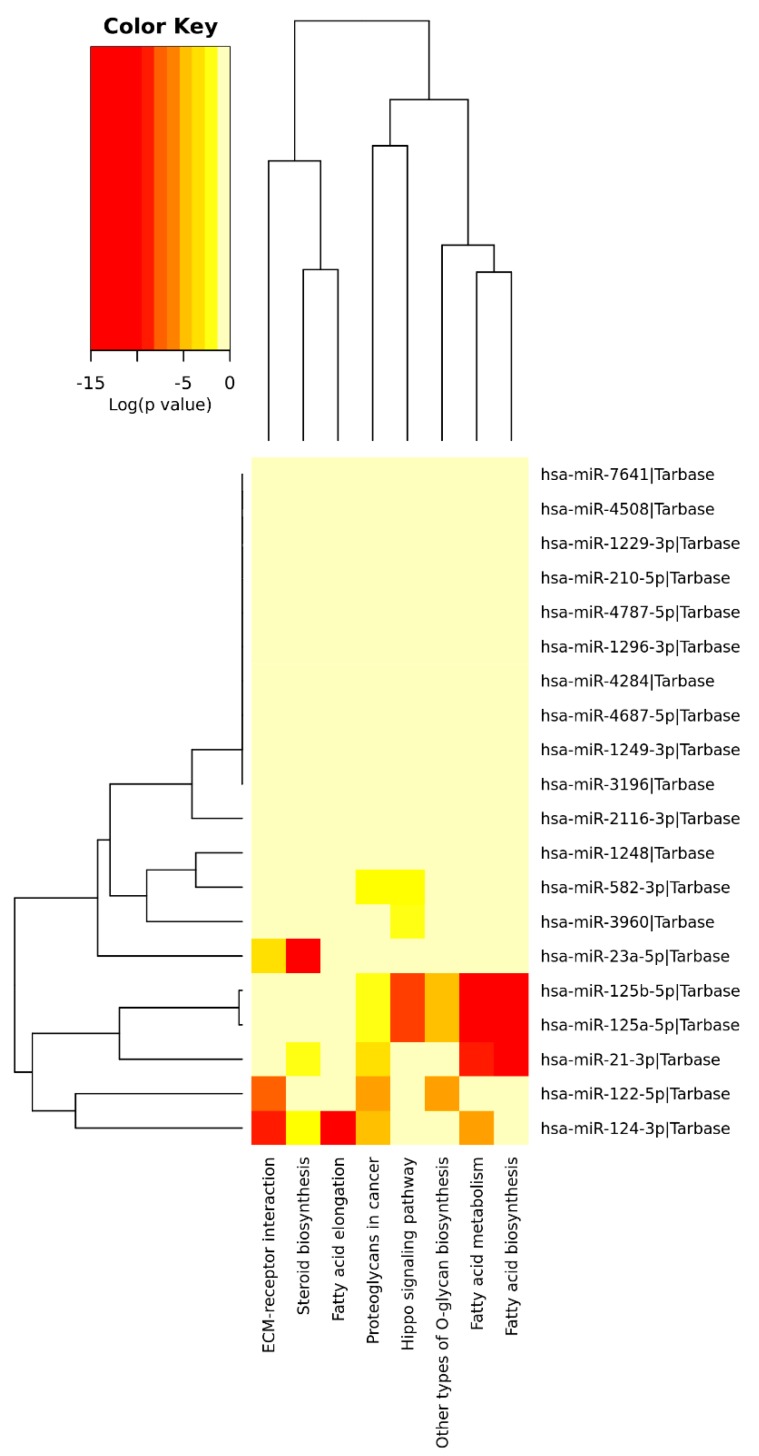
A heatmap viewer of a KEGG pathways union analysis with Tarbase: Overrepresented pathways by SDE miRNA targets between the CHC and SC groups with respect to HC.

Targets from miRNAs associated with HCV+ exposed individuals were explored for enrichment in known KEGG pathways. Among the 21 SDE miRNAs identified between CHC patients and SC individuals with respect to HC, eight significantly enriched pathways (*p* < 0.05) were identified based on experimentally validated data (Figure 6, Table 2). The most significant pathways were those related to fatty acid metabolism (hsa00061, hsa01212, and hsa00062), where miRNAs hsa-miR-21-3p, hsa-miR-125a-5p, hsa-miR-125b-5p, and hsa-miR-124-3p have key roles. All these miRNAs, except miR-21-3p and miR-124-3p, were downregulated in CHC and SC regarding HC. Other significant pathways targeted by these miRNAs were (a) the Hippo signaling pathway (hsa04390), identified by 27 targeted genes of 4 SDE miRNAs (hsa-miR-125a-5p, hsa-miR-125b-5p, hsa-miR-582-3p, and hsa-miR-3960); (b) the extracellular matrix (ECM) interaction pathway (hsa04512), which is represented by 24 genes targeted by 3 SDE miRNAs (hsa-miR-23a-5p, hsa-miR-122-5p, and hsa-miR-124-3p); (c) two proteoglycans-related pathways (hsa05205, hsa00514), represented in total by 105 genes targeted by 6 SDE miRNAs (hsa-miR-21-3p, hsa-miR-122-5p, hsa-miR-124-3p, hsa-miR-125a-5p, hsa-miR-125b-5p, and hsa-miR-582-3p); and (d) the steroid biosynthesis (hsa00100), denoted by 5 targeted genes of 3 SDE miRNAs (hsa-miR-21-3p, hsa-miR-23a-5p, and hsa-miR-124-3p).

### 3.5. Network of miRNAs and their targeted genes

**Figure 7:**
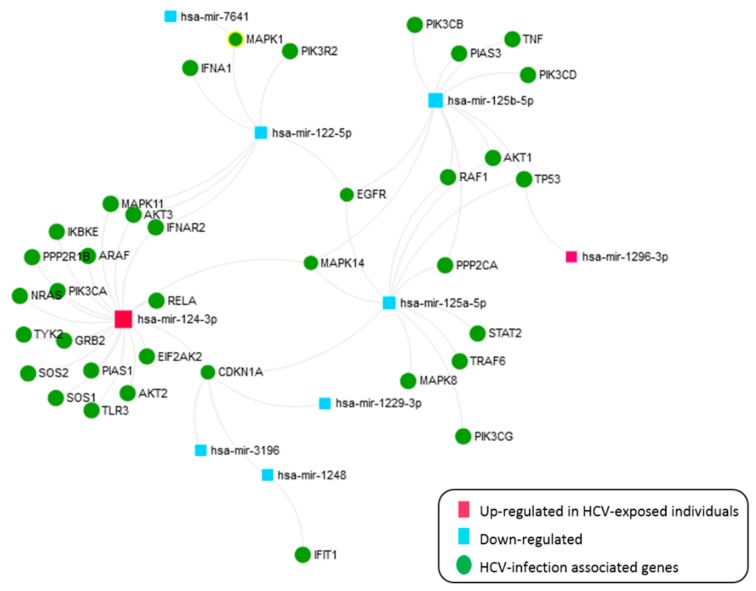
An interactive network of SDE miRNAs in CHC patients and SC individuals versus HC, corresponding to the HCV KEGG pathway.

The 21 SDE miRNAs were analyzed with miRNEt (Figure 7). This analysis identified nine miRNAs (miR-124-3p and miR-1296-3p upregulated and miR-125a/b-3p, miR-122-5p, miR1229-3p, miR-1248, miR-3196, and miR-7641 downregulated in either CHC or SC individuals) with 39 target gene interactions corresponding to the HCV KEGG pathway. Thus, the miR-124-3p is critical in this pathway, as they directly target 18 HCV infection-related genes. Therefore, our results suggest a dysregulation of genes associated with HCV infection, either in an active or previous acute HCV status.

## 4. Discussion

Our major purpose was to find a similar deregulation of miRNAs in both CHC patients and SC patients with respect to the HC group. Therefore, a spontaneous clarification of HCV during an acute infection leads to molecular changes that can be observed later in the miRNA profile of PBMCs.

HCV infection in hepatic cells triggers an extensive dysregulation of host miRNAs to positively or negatively regulate its life cycle through directly targeting its genome and genes associated with cellular signaling pathways [25,26]. Our study is the first report showing a disruption of miRNA expressions in PBMCs of HCV-previously exposed patients, where this disruption persists after an HCV spontaneous resolution.

Patients for the present study were recruited just before the inclusion of the new direct active antivirals (DAAs) in the Spanish public healthcare system. At that time, only patients with advance fibrosis were treated with the classic interferon and ribavirin treatment, while the rest of individuals wait to be treated with DAAs. For this reason, we could recruit CHC naïve patients with a long time of infection (up to 38 years). Although all CHC patients showed no advanced fibrosis, the values of GOT, GPT, GGT, and APRI were significantly higher than in SC individuals. This different liver enzyme profile strengthens our claim on miRNA profile similarity in SC and CHC patients.

Our findings were reached through three different methods of statistical analysis since there is no clear consensus on the appropriate normalization method to be used in miRNA sequencing data. The choice of the normalization method is the primary factor that affects the results of a high-throughput differential expression analysis, and this will inevitably impact the downstream analysis [27]. For this reason, we have only selected those SDE miRNAs identified by the three methods to limit false positives results and to secure the validity of our findings. This method of analysis, together with the use of a large cohort of patients for a high-throughput analysis, strengthen the confidence of our results.

We identified a signature of 21 SDE miRNAs disrupted in CHC and SC individuals, with respect to HC. These miRNAs were dysregulated with similar fold changes and the same direction in both comparisons, which indicates that either a previous acute or active chronic HCV infection similarly disrupts miRNA profiles of PBMCs over time. These 21 SDE miRNAs can differentiate HCV-exposed (SC and CHC) from HC without classification error, being that hsa-miR-21-3p is the variable with the best performance. This miRNA is upregulated in our HCV-exposed group of patients, which is in accordance with previous in vitro studies [28]. HCV induces hsa-miR-21-3p, which negatively regulates IFN-alpha signaling, contributing thus to an evasion of the host immune system. For this reason, Chen et al. stated that has-miR-21-3p might be a potential therapeutic target for antiviral intervention.

Five out of 21 SDE miRNAs have not been previously detected in PBMCs (hsa-miR-7641, hsa-miR-1248, hsa-miR-1229-3p, hsa-miR-1296-3p, and hsa-miR-4284). Only the hsa-miR-7641 has shown a previous relation to HCV infections [29], where its expression was downregulated in severe versus mild CHC. Our data also showed a downregulation in HCV-exposed patients versus HC. On the other hand, two miRNAs (hsa-miR-1296-3p and hsa-miR-4284) have shown relations to chronic hepatitis B (CHB). The hsa-miR-1296-3p is expressed in hepatocellular carcinoma of chronic hepatitis B infected patients [30], while plasma levels of the hsa-miR-4284 seem to predict early virological response to interferon alpha therapy in CHB patients [30,31], and it is upregulated in recurrent hepatocellular carcinoma [32].

To the best of our knowledge, this is the first study reporting the miRNA profile of SC patients. Some others have slightly addressed differences between CHC and HC individuals, but no high-throughput assays have been performed. The widely studied hsa-miR-122 was significantly downregulated in HCV-exposed patients (CHC and SC) in our data set. Despite differences in the study design, similar results have been previously observed in either liver or PBMCs of CHC patients [33]. The expression profiling of PBMC associated miRNAs was also compared between responders and non-responders to PEG-IFN/RBV antiviral therapy. His and colleges identified that circulating PBMC associated miR-125b was a predictive marker for SVR with significantly reduced levels of miR-125b in responders versus non-responders [34], but no control group was used. Our results indicate that this miRNA is downregulated in HCV-exposed. Matsuura et al. [29] analyzed miRNA expression in CHC patients compared to HC, but they performed this analysis in plasma and extracellular vesicles not in PBMCs. Also, unlike our patients, in Matsuura et al., CHC patients showed mild and advanced fibrosis. We have compared our results with this study, and only two miRNAs are common, hsa-miR-3960 and hsa-miR-4787-5p.

We explored all published data sets of HCV infections to validate our data. Unfortunately, as no similar studies are freely available, we could not validate the classification performance of this 21 miRNA in other data sets.

### 4.1. Lipid Metabolism Disruption in HCV-Exposed Patients

The enriched pathways analysis of the targets regulated by the HCV signature of 21 SDE miRNAs showed that the fatty acid metabolisms pathways were the most significant, where hsa-miR-21-3p and miR- 124-3p were upregulated while hsa-miR-125a-5p and hsa-miR-125b-5p were downregulated. These miRNAs have been identified as disrupted in HCV-infected hepatocytes, but this is the first report showing a lipid metabolism-related miRNAs disruption in PBMCs of HCV-exposed individuals.

Metabolic pathways related to cholesterol and lipoproteins biosynthesis have been shown to play a critical role in an HCV life cycle and are essential for HCV uptake, formation, and replication of new viral elements [25,35]. In this sense, HCV hijacks and manipulates fatty acid flux to create specific lipid-enriched microenvironments to promote its life cycle [36,37]. This deregulation directly affects the innate antiviral response, liver disease progression, and the response to antiviral therapies [38]. Emerging evidence demonstrates that miRNAs are critical regulators of lipid biosynthesis, fatty acid oxidation, and lipoprotein formation and secretion [39]. Also, not only is the deregulation of lipid metabolism during an HCV infection limited to hepatocytes but PBMCs may reflect the alterations of intracellular pathways occurring during HCV and also HBV liver diseases [17].

In this setting, we have explored the lipid profile of SC and CHC patients, but similar to miRNA results, both profiles were similar with no statistically significant differences among them. Similar results were found in a small study on CHC and SC Mexican patients [40], where both groups showed similarities in lipid profile but differences in liver enzymes and cytokines. However, a lipidomic analysis on the serum of HCV-resistant cases showed clear differences from HCV antibody-positive individuals, even those who clear viremia either spontaneously or after antiviral therapy [41].

MiR-21-3p was upregulated in HCV-exposed subjects (CHC and SC groups). This miRNA is also upregulated (up to 21-fold) in HCV in vitro infection [28], where its increase allows HCV to evade the immune surveillance system. This miRNA targets two crucial factors in the TLR signaling pathway, *myeloid differentiation factor 8* (*MyD88*) and *interleukin-1 receptor-associated kinase 1* (*IRAK1*), which are both involved in HCV-induced type I interferon production. Therefore, miR-21 is considered as a potential therapeutic target for HCV antiviral therapy as it negatively regulates interferon-alpha signaling. The miR-21 expression has also been positively correlated with the fibrotic stage and could lead to increased fibrogenesis by targeting genes belonging to the transforming growth factor (TGF)-β signaling pathway [42]. Regarding miR-21 effects on fatty acid metabolism pathway, this molecule targets some essential genes such as *insulin-like growth factor binding protein 3* (*IGFBP3*), *fatty acid-binding protein 7* (*FABP7*), and *peroxisome proliferator-activated receptor alpha (PPARα)* [39], which regulate cell growth, intracellular trafficking of fatty acids, and and gene transcription of fatty acid metabolism, respectively.

MiR-124-3p, a key regulator of fatty acid oxidation and triglyceride (TG) metabolism [43], is also involved in Treg cell development during an HCV infection [44]. Through the targeting of multiple genes, miR-124-3p produces a cooperative inhibitory effect on lipid catabolism, leading to TG accumulation in human hepatoma Huh7.5 cells. Our data show an upregulation of miR-124-3p in PBMCs during and after an HCV infection, which may be related to a lipid accumulation to facilitate HCV replication. This miRNA was one of the most important in HCV-exposed vs. non-exposed individuals in the PLS-DA.

hsa-miR-125a-5p and 125b-5p were both detected as downregulated in PBMCs in the CHC and SC groups. These miRNAs are encoded by genes located at different chromosomes but share closely related mature sequences and seed family. Thus, both miRNAs target similar genes and regulate related pathways, such as the tumor necrosis factor alpha-induced protein 3 (TNFAIP3, A20), a negative regulator of the NF-κB pathway [45] that has been previously associated with liver fibrosis and inflammation in an HCV infection [46]. Bala et al. also found that miR-125b-5p was downregulated in monocytes of HCV chronic patients compared to healthy controls [47]. Regarding lipid metabolism, miR-125a-5p negatively regulates the *scavenger receptor class B type I* (*SR-BI*), which encodes for the receptor that captures the cholesterol esters from HDL particles for steroidogenesis, mainly in hepatocytes [48]. Therefore, the downregulation of miR-125a-5p will produce a higher expression of *SR-BI* and, consequently, a higher uptake of cholesterol in PBMCs, which could facilitate HCV infection. On the other hand, miR-125b-5p seems to play a vital role in lipogenesis by targeting the *stearoyl-CoA desaturase-1 (SCD-1)* gene, which encodes for a key enzyme in lipogenesis in which inhibition impairs lipid synthesis [49]. Thus, the downregulation of this miRNA in the context of an HCV infection could be related to an increase of fatty acid deposits within infected cells, which would again benefit the HCV viral cycle.

### 4.2. Additional Disrupted Pathways in PBMCs After HCV Infection

Further signaling pathways were significantly identified in a functional enrichment analysis of the 21 SDE miRNAs. The Hippo signaling pathway is an evolutionarily conserved pathway that controls organ size by regulating cell growth and proliferation. Previous studies have identified the inactivation of this pathway in the development of hepatocellular carcinoma (HCC) [50,51,52], and it is interconnected with other crucial signaling cascades such as TGF-beta and Wnt growth factors. Interestingly, this pathway has a key role in pathogen-infected cells by regulating innate antiviral immunity [53]. Also, some virus and bacteria can exploit hippo signaling to enhance pathogenicity by evading host innate immunity. Our data indicate that this pathway is disrupted in PBMCs of HCV-exposed individuals (CHC and SC groups) by the upregulation of hsa-miR-125a-5p, hsa-miR-125b-5p, and hsa-miR-3960 and by the downregulation of hsa-miR-582-3p.

On the other hand, the extracellular matrix (ECM) interaction pathway (hsa04512) seems to be dysregulated in PBMCs in active and previous HCV exposure. This pathway is involved in liver fibrosis development, which is triggered by an HCV infection through the activation of hepatic stellate cells (HSCs) [54]. The ECM is involved in the regulation of the interferon signaling pathway and HCV-RNA replication [55]. PBMCs contribute to systemic and regional inflammation and matrix remodeling in some model diseases, such as cardiac disease [56] and obesity [57]. Therefore, it is reasonable that PBMCs from patients exposed to HCV show a disruption of the ECM pathway as a consequence of HCV presence within these cells [58].

Proteoglycans-related pathways (hsa05205 and hsa00514) are also disrupted. These pathways have been described as responsible for the tissue-specific tropism of an HCV infection [58], and some studies have identified the involvement of proteoglycans, such as heparan sulfate, in HCV attachment and postattachment steps [59]. Three SDE miRNAs were upregulated in HCV-exposed patients (miR-21-3p, miR-124-3p, and miR-582-3p) and three were downregulated (miR-125a-5p, miR-125b-5p, and miR-122-5p). Therefore, HCV infections could be modifying specific cell-surface proteoglycans of infected cells through miRNA disruption to facilitate HCV attachment.

Also, we identified the steroid biosynthesis pathway (hsa00100) as dysregulated in HCV-exposed individuals. A previous expression analysis of in vitro experiment in naïve, HCV-infected, and cured cells showed that the steroid biosynthesis is one of the significantly altered pathways, suggesting that, among others, it is required for an efficient HCV replication [60]. Steroid enzymes are responsible for the biosynthesis of various steroid hormones such as glucocorticoids, mineralocorticoids, progestins, androgens, and estrogens from cholesterol. This closely related pathway to lipid and cholesterol metabolism has also an essential role in immune cell-mediated metabolic regulation and immunomodulation [61]. Thus, our data indicate that, during chronic and after a spontaneously resolved HCV infection, PBMCs show an upregulation of miR-21-3p (which target *DHCR4* and *NSDHL*) and miR-124-3p (which targets *SC5D, DHCR24, LIPA,* and *FAXDC2*) and the downregulation of miR-23a-5p (which also targets *DHCR24*). This complex deregulation of cholesterol and steroid biosynthesis enzymes seems to be modified by HCV infections to promote a permissive HCV infective environment.

### 4.3. HCV-Related Genes

We have analyzed the implication of the 21 SDE miRNAs in targeting host genes related to HCV infection, and nine out of 21 SDE miRNAs were identified. The most relevant miRNA in this analysis was the has-miR-124-3p, which was upregulated in HCV-exposed individuals, and it targets 18 HCV infection-related genes. HCV infection requires multiple host signaling pathways to successfully infect the host cell, and it has developed strategies to disrupt the induction of IFN and cytokine pathways, favoring viral propagation and presumably HCV chronic infections [62]. Our results suggest that the subverts of miRNAs is an additional strategy of HCV to maintain infection [8] and to establish an antiviral stage. Thus, the upregulation of hsa-miR-124 in PBMCs by HCV will downregulate IFN production at different pathway levels, such as the IFN alpha/beta pathway [63], the PI3K/AKT signaling pathway [64], and the MAPK/ERK cascade which is used by HCV for viral propagation [65]. Here, we review the most relevant targets of this miRNA and their biological functions.

HCV interacts with components of the IFN alpha/beta pathway to inhibit IFN production, and the hsa-miR-124-3p can target seven members of this signalling pathway: the *toll-like receptor 3* (*TLR3*), the *inhibitor of nuclear factor kappa B kinase subunit epsilon* (*IKBKE*), *the eukaryotic translation initiation factor 2 alpha kinase 2* (*EIF2AK2*), the *interferon-alpha/beta receptor 2* (*IFNAR2*), the *tyrosine kinase 2* (*TYK2*), the *protein inhibitor of activated STAT 1* (*PIAS1*), and *RELA proto-oncogene* (*RELA*). Thus, the overexpression of has-miR-124-3p may act as an additional strategy to disrupt the host antiviral state.

HCV also activates PI3K-Akt signaling to enhance entry, replication, and translation [66,67]. Three main factors of this pathway targeted by has-miR-124 are the *phosphatidylinositol-4,5-bisphosphate 3-kinase catalytic subunit alpha* (*PIK3CA*) and the *AKT serine/threonine kinase 2* (*AKT2*) and *3* (*AKT3*). The assault by HCV on this pathway that regulates cell growth and metabolism probably plays an important role in HCV pathogenesis.

HCV also modulates the MAPK/ERK cascade for its own propagation [65]. Eight genes within this pathway can be targeted by the hsa-miR-124-3p: *the growth factor receptor-bound protein 2* (*GRB2*), *SOS Ras/Rho guanine nucleotide exchange factor 1* (*SOS1*) and *2* (*SOS2*), *A-Raf proto-oncogene, cytosolic serine/threonine kinase* (*ARAF*), the *GTPase neuroblastoma RAS proto-oncogene* (*NRAS*) [68], the *mitogen-activated protein kinase 11* (*MAPK11*), the *protein phosphatase 2 scaffold subunit Abeta* (*PPP2R1B*), and the *cyclin-dependent kinase inhibitor 1A* (*CDKN1A*) also known as p21. Different viruses such as HCV interact with this pathway to specifically subvert key survival pathways for the host [69]. Therefore, the upregulation of hsa-miR-124 by an HCV infection will repress the RAS-MAPK signaling pathway at different levels to ultimately regulate HCV RNA replication.

### 4.4. HCV Spontaneous Clarification

The common miRNA profile between SC and CHC suggests that an HCV infection promotes molecular alterations that will last long after an HCV spontaneous eradication. Our cohort with 36 spontaneous clarifiers was recruited from routine controls, where anti-HCV antibodies were detected a long time after infection (up to 47 years). There is not much information on the dynamics of antibodies against HCV in patients who eliminated an HCV infection [70,71]. Most of the studies have been focused on the anti-HCV antibody levels of CHC patients after IFN therapy, but scarce studies have analyzed the anti-HCV dynamics after a spontaneous clarification [4,72]. Takaki et al. reported that HCV-specific antibodies were lost in some patients 18–20 years after recovery from an acute-phase of HCV infection [4], while positive HCV antibodies can be detected even 18 years after inoculation, as it has been previously identified in a cohort of HCV spontaneously resolved Irish women [73]. These findings suggest that SC may be underestimated in the general population, as antibodies anti-HCV can be lost over time in some individuals.

Several comorbidities such as steatosis and nonalcoholic steatohepatitis had been previously observed at a higher frequency in these Irish patients [74]. They analyzed biopsies from HCV PCR-negative individuals with the modified histological activity index (HAI), and a high proportion of steatosis was reported, as well as mild inflammation and steatohepatitis. Then, the authors suggested a “cleared past exposure” in the liver of spontaneously resolved patients.

Our results indicate that the SC group suffer from PBMCs-fatty acid metabolism disruption, which could reflect liver status. Previous evidence supports this idea as the fatty liver is predominant in HCV patients after the achievement of sustained virological response [75] and the HCV cure does not lead to complete immunological restitution [76]. A prior history of spontaneously resolved HCV infections may promote hepatic injury in those patients with non-alcoholic fatty liver disease (NAFLD) or alcohol-related liver disease, but this field remains unexplored. Therefore, an additional follow-up of previously HCV-exposed patients should be carried out to explore putative future complications such as liver diseases, among others.

Therefore, the availability of biomarkers to identify HCV-exposed individuals is essential. Hence, our 21 miRNA signature could be used to identify SC patients with undetectable antibodies against HCV.

Future studies will be required to delve into the role of these 21 SDE miRNA identified during and after an HCV infection and their implication in the immune restoration of PBMCs.

Finally, some additional considerations should be taken into account to interpret our results properly. The lipid profile of HC could not be recorded, so we could not analyze the differences compared to the HC and CHC groups. The immune host-related background has a strong influence on the spontaneous clearance of an HCV infection, which variates with ethnicity and gender. Our study has been carried out entirely in Caucasian patients, and it would be necessary to perform an independent replication of this study for different ethnic groups. Also, our cohort of patients was sex balanced, with the same proportion of males and females. This design allows us to remove gender bias, as it has been widely described that females clarify HCV in a higher proportion than men. The protective allele of IFNL4 (rs12979860) showed a higher frequency in SC patients, which has been strongly associated with a spontaneous HCV clearance irrespective of the HCV-infected genotype, and reflects the spontaneous resolution genetic background of these patients.

## 5. Conclusions

Our data indicate that HCV spontaneous resolved individuals and HCV chronic patients showed a similar disruption of miRNA profile at PBMCs with respect to healthy controls. This disruption is reflected in a fingerprint of 21 miRNAs that correctly differentiates HCV-exposed individuals from non-exposed. These miRNAs mainly regulates fatty acid metabolism. Therefore, a spontaneous clarification of HCV during an acute infection leads to molecular changes that can be subsequently observed on the miRNA profile of PBMCs.

## Figures and Tables

**Table 1:** HCV-related clinical and epidemiological characteristics of 96 patients with miRNA sequencing data.

Patients Characteristic	All Patients	CHR	SC	HD	*p* Value
No.	96	32	32	32	
Gender (male %)	48 (50%)	16 (50%)	16 (50%)	16 (50%)	1.000
Age at recruitment (years)	50.5 (45.0; 59.0)	50.5 (43.0; 62.2)	54.5 (47.0; 60.5)	48.5 (42.5; 56.2)	0.171
Time of HCV infection (years) (*n* = 32)	-	24.0 (17.5; 34.0)		-	-
Time of HCV spontaneous clearance (years) (*n* = 23)	-	-	3 (2; 13)	-	-
Transmission route (No.)					<0.001
IDUs	19 (19.8%)	13 (40.6%)	6 (18.8%)	-	
Transfusion	8 (8.3%)	6 (18.8%)	2 (6.3%)	-	
Medical Procedure	6 (6.3%)	5 (15.6%)	1(3.1%)	-	
Unknown	63 (65.6%)	8 (25.0%)	23 (71.9%)	-	
Fibrosis stage (No.)	41	32	9		0.378
F0	6 (14.6%)	5 (15.6%)	1 (11.1%)	-	
F1	32 (78%)	25 (78.1%)	7 (77.8%)	-	
F2	1 (2.4%)	0 (0%)	1 (11.1%)	-	
HCV viral load (UI/mL) × 106	-	1.5 (0.6; 3.3)	-		
HCV genotype (*n* = 32)					-
1	-	22 (68.8%)	-		
2	-	3 (9.4%)	-		
3	-	1 (3.1%)	-		
4	-	6 (18.8%)	-		
IFNL4 (IL28B) SNP (*n* = 95)					0.004
CC (favourable)	42 (44.2%)	6 (18.8%)	20 (64.5%)	16 (50%)	
CT	41 (43.2%)	20 (62.5%)	7 (22.6%)	14 (43.8%)	
TT	12 (12.6%)	6 (18.8%)	4 (12.9%)	2 (6.3%)	

Values expressed as absolute numbers (%) and median (percentile 25; percentile 75). *p*-values were estimated by nonparametric Kruskal–Wallis test and Mann–Whitney for continuous variables and by the Chi-square test for categorical variables. Statistically significant differences are shown in bold. Abbreviation: HCV, hepatitis C virus; BMI, body mass index; IDUs, intravenous drug users; LS, liver stiffness; IFNL4, interferon lambda 4 (gene/pseudogene).

**Table 2:** Clinical characteristics of metabolic and liver status.

Patients characteristic	All Patients	C	SC	HC	*p* Value
Total number	96	32	32	32	
Weight (kg) (*n* = 70)	71.8 (61.1; 80.0)	65.0 (58.0; 75.3)	73.3 (64.2; 87.5)	75.8 (64.0; 88.2)	0.274
BMI (*n* = 64)	24.9 (22.2; 27.5)	24.8 (21.6; 26.7)	24.9 (21.6; 28.4)	25.2 (22.8; 29.0)	0.655
Glucose (mg/dL) (*n* = 64)	89.0 (80.2; 102.5)	89. 0 (80.2; 108.5)	88 (80.5; 102.5)	-	0.697
Glucose ≥110 mg/dl	13 (20.3%)	8 (25.0%)	5 (15.6%)	-	0.351
Insulin (mlU/L) (*n* = 23)	-	8.5 (5.5; 13.9)	-	-	
HOMA-IR (*n* = 23)	-	1.75 (1; 3.2)	-	-	
Lipid profile					
Total cholesterol mg/dL (*n* = 63)	186.0 (161.0; 207.0)	177.5 (156.25; 206.2)	192.0 (173.0; 207.0)	-	0.132
TC ≥200 mg/dL	19 (30.2%)	9 (28.1%)	10 (32.3%)	-	0.721
LDL (*n* = 56)	105.0 (85.0; 125.0)	104.5 (85.0; 131.2)	107.0 (89.0; 116.0)	-	0.918
LDL ≥130 mg/dL	10 (18.2%)	8 (25.0%)	2 (8.7%)	-	0.122
HDL mg/dL (*n* = 55)	57.0 (46.0; 65.0)	57.5 (46.2; 65.0)	55.0 (46.0; 66.0)	-	0.898
TG (*n* = 56)	91.5 (66.7; 149.0)	92.0 (61.0; 152.0)	91.0 (72.0; 137.0)	-	0.676
TG ≥200 mg/dL	3 (5.4%)	2 (7.4%)	1 (4.3%)	-	0.737
Homocysteine (µmol/L) (*n* = 22)	13.4(10.8; 16.3)	13.7 (12.1; 16.6)	12.0 (10.2; 16.1)	-	0.553
High level (>15)	7 (11.1%)	3 (9.7%)	4 (12.5%)	-	0.722
LDL/HDL (*n* = 55)	1.96 (1.5; 2.3)	1.8 (1.4; 2.5)	1.8 (1.5; 2.3)	-	0.645
AI (TC/HDL) (*n* = 41)	3.1 (2.8; 3.8)	3.1 (2.7; 3.9)	3.3 (3.0; 3.8)	-	0.588
Low risk	38 (39.6%)	24 (75.0%)	14 (43.8%)	-	0.903
Moderate risk	3 (3.1%)	2 (6.3%)	1 (3.1%)	-	0.903
AIP (Log(TG/HDL)]) (*n* = 41)	0.19 (0.07; 0.47)	0.20 (0.02; 0.49)	0.18 (0.08; 0.35)	-	0.948
AIP High risk (>0.21)	19 (45.2%)	13(48.1%)	6 (40%)	-	0.611
LCI (TC*TG*LDL)/HDL × 103 (*n* = 42)	31.4 (22.2; 50.9)	30.7 (14.5; 58.3)	34.8 (24.4; 36.8)	-	0.948
Biochemical parameters of liver function					
GOT (*n* = 63)	34.0 (24.0; 42.0)	37.0 (31.2; 45.0)	24.0 (18.0; 37.0)	-	0.002
GOT ≥40 mg/dL	14 (22.2%)	9 (28.1%)	5 (16.1%)	-	0.252
GPT (*n* = 64)	33.0 (21.5; 52.5)	41.0 (32.2; 59.7)	25.5 (17.2; 37.5)	-	<0.001
GPT ≥40 mg/dL	25 (39.1%)	18 (56.3%)	7 (21.9%)	-	0.005
GGT (*n* = 62)	32.5 (21.7; 60.2)	40.0 (25.0; 69.0)	24.0 (15.0; 57.0)	-	0.039
GGT ≥50 mg/dL	22 (22.9%)	12 (12.5%)	10 (31.25)	-	0.596
ALP (*n* = 62)	74.0 (58.0; 85.7)	75.5 (57.7; 89.2)	73.5 (58.2; 83.5)	-	0.472
TB (*n* = 63)	0.6 (0.5; 0.8)	0.6 (0.5; 0.9)	0.5 (0.4; 7.0)	-	0.116
Albumin (*n* = 45)	4.4 (4.2; 4.6)	4.4 (4.2; 4.6)	4.3 (4.2; 4.5)	-	0.465
APRI (*n* = 57)	0.4 (0.3; 0.6)	0.5 (0.3; 0.7)	0.3 (0.3; 0.4)	-	0.032
FIB-4 (*n* = 55)	1.4 (1.0; 2.0)	1.4 (1.0; 1.8)	1.4 (1.1; 2.0)	-	0.748

Values expressed as absolute numbers (%) and median (percentile 25; percentile 75). p-values were estimated by nonparametric Kruskal–Wallis test and Mann–Whitney for continuous variables and by the Chi-square test for categorical variables. Statistically significant differences are shown in bold. BMI, body mass index; TC, total cholesterol; LDL, low density lipoprotein; TG, triglycerides; HDL, high density lipoprotein; AI, atherogenic index, cut-off values were: low risk <5% for men and <4.5% for women, moderate risk 5–9 for men and 4.5–7 for women; AIP, atherogenic index for plasma considering high risk AIP > 0.21; LCI, lipoprotein combine index defined as the ratio of TC*TG*LDL to HDL-C; GOT, glutamate oxaloacetate transaminase; GPT, glutamic-pyruvic transaminase; GGT, gamma-glutamyltransferase; ALP, alkaline phosphatase; TB, total bilirubin; APRI, AST to platelet ratio index; FIB-4, fibrosis-4 index for liver fibrosis.

## Supplementary Materials

The following are available online at https://www.mdpi.com/2077-0383/8/6/849/s1, Figure S1: miRNA validation, Table S1: de novo miRNAs, Table S2: SDE miRNAs summary, Table S3: SDE miRNAs between SC and HC, Table S4: SDE miRNAs between CHC and HC, Table S5: SDE miRNAs between CHC and SC, Table S6: miRNA validation primers, Table S7: Validation cohort for qRT-PCR, and Table S8: miRNA validation by qRT-PCR.
