# Supplementary material for: MicroRNA Profile of HCV Spontaneous Clarified Individuals, Denotes Previous HCV Infection
Source: J Clin Med. 2019 Jun 14;8(6):849. doi: 10.3390/jcm8060849 (PMC6617112; doi:10.3390/jcm8060849)
Supplement: Supplementary file 1 [file jcm-08-00849-s001.pdf]

Table S1: miRNAs de novo.

Table S2. The number of significant differentially expressed (SDE) miRNAs between each group of patients, estimated by three methods of analysis (DESeq, edgeR, and NOISeq).

Table S3. Significant differentially expressed (SDE) miRNAs between an HCV spontaneous clarifier subjects and healthy donors.

Table S4. Significant differentially expressed (SDE) miRNAs between HCV+ chronic patients and healthy donors.

Table S5. Significant differentially expressed (SDE) miRNAs between HCV+ chronic patients and spontaneous clarifiers subjects.

Table S6. The validation cohort for qRT-PCR.

Table S7. hsa-miR-21-3p and has-miR-23a-5p expression by qRT-PCR in a set of 66 different patients.

Figure S1: miRNA validation.

Supplementary Materials and Methods

Table S1. The 137 de novo miRNAs identified by miRDeep2 that fulfilled the filtering criteria (see the Materials and Methods section).

provisional id	miRDeep2 score	total read count	consensus mature sequence	precursor coordinate
chr8_19615	55,921.6	109,687	acccugcucgcugcacca	chr8:55729558..55729627:+
chr7_18080	12,273.4	24,073	ucggcuguguaucucugugucc	chr7:142474536..142474590:+
chr1_2076	5602.3	10,990	aguugggagagcauuagacuga	chr1:212846839..212846904:+
chr3_8150	1288.4	2525	ggacgaaauccaagcgcagcug	chr3:49020632..49020697:+
chr9_22161	1007.9	1974	agagcaaagaaccugagugcc	chr9:42435995..42436063:-
chr9_21296	1006.9	1972	agagcaaagaaccugagugcc	chr9:66697258..66697326:+
chr9_22135	1006.9	1972	agagcaaagaaccugagugcc	chr9:39679635..39679703:-
chr9_21274	1006.9	1972	agagcaaagaaccugagugcc	chr9:62662727..62662795:+
chr10_23371	863.5	1692	ccacgaggaagagagg	chr10:80486623..80486703:+
chr18_38087	701.6	1376	cuucgaaagcggcuucggcu	chr18:3448120..3448161:-
chr5_14270	690.9	1353	uuugacuugaacucauuccccagg	chr5:177505109..177505198:-
chr1_1401	666.7	1305	caagagccuggaacugcacca	chr1:148530902..148530961:+
chr1_3464	666.7	1305	caagagccuggaacugcacca	chr1:120842640..120842699:-
chr1_1293	666.7	1305	caagagccuggaacugcacca	chr1:145289411..145289470:+
chr1_3688	666.7	1305	caagagccuggaacugcacca	chr1:149556790..149556849:-
chr1_1257	650.7	1274	caagagccuggaacugcacca	chr1:144420900..144420960:+
chr3_7834	608	1185	aaaaguaaucgcggucuuugcc	chr3:4451433..4451491:+
chr16_33771	604.1	1176	aaaaguaaucgugguuuuugcc	chr16:47674361..47674418:+
chr10_24527	598.4	1172	cacaacugagcaucacagccuga	chr10:122416979..122417050:-
chr22_43283	571.5	1111	aaaaguaaucgcggucuuugcc	chr22:36340173..36340232:-
chr22_42782	569.5	1107	aaaaguaaucgcggucuuugcc	chr22:36340176..36340235:+
chr19_38982	558.2	1092	gugugugcaccugugucugucugu	chr19:18284682..18284742:+
chr12_29021	557.1	1091	aauggaggaggaggaag	chr12:113875917..113875951:-
chr12_29022	556.9	1091	aauggaggaggaggaag	chr12:113875917..113875951:-
chrX_44362	556.3	1098	aagguguagcccaugag	chrX:143430647..143430692:+
chr3_8235	404.9	792	aacaggccuugcucugcucacaga	chr3:52523355..52523427:+
chr2_6109	383.4	751	ccagcugccucuccuccaucg	chr2:233288737..233288798:+
chr3_9873	371.7	726	uguuuagcauccuguagccugc	chr3:143783124..143783180:-
chr16_34569	356.9	696	cggcggcuccagggaccuggcg	chr16:30571617..30571677:-
chr10_23092	355.5	694	uggcuggcugcuccgggcacug	chr10:43471237..43471296:+
chr18_37981	343.1	672	acuggcaucgugauggacu	chr18:62442386..62442472:+
chr19_39642	294.5	575	uuaguggcuccccucugccugca	chr19:57439110..57439173:+
chr12_28785	286.7	553	ucuggcuccuuucuaaucacu	chr12:80906573..80906631:-
chr8_20578	274.4	535	uuauccuccaguagacuaggga	chr8:98393666..98393725:-
chr1_1467	270.3	528	ugaggccgagaaggcaaccgcga	chr1:150234737..150234790:+
chr1_3204	269.1	526	cagggccuggaacuccaauggga	chr1:77960393..77960454:-
chr14_31644	261	511	uugcucugcucucccuuguacu	chr14:95533806..95533866:-
chr2_6327	233.9	457	aauccucacuuugaauccauguu	chr2:16431472..16431532:-
chr11_25739	232.2	453	cccagagaaggcugcuccucacca	chr11:121482469..121482529:+
chr11_25835	224	443	uguguagggcauggcucagacu	chr11:133128476..133128542:+
chr19_38809	219	428	cuuccccacccucuccugcagc	chr19:12952628..12952689:+
chr5_13708	209.5	408	cgccgcuuucugggcucgcuca	chr5:108748634..108748691:-
chr16_34282	195.9	383	acaugugucugugucugcccagc	chr16:3548217..3548271:-
chr7_17373	195.1	380	caacagaacccagagagucagccu	chr7:37784895..37784962:+
chr13_29287	190.1	363	uaugugccuaguggcugcugucu	chr13:26685333..26685399:+
chr14_31787	189.7	370	uggcugagcugagaaccacugugc	chr14:105865527..105865584:-
chr9_21973	187.5	365	gcgggcgggcgggcgg	chr9:21031447..21031479:-
chr2_6060	186.6	375	auuuugauuuucaguac	chr2:228355021..228355068:+
chr19_39102	177	345	ucaugucugaaccaaugagagc	chr19:35161525..35161585:+
chr19_38639	171.4	334	uaucugcuguuguccccucagg	chr19:7468769..7468825:+
chr4_11295	166.7	324	agaugggggagaacucaauccu	chr4:48220121..48220183:-
chr1_2400	166.2	323	cuggccugucuccugcccagg	chr1:1205491..1205549:-
chr3_8223	159.2	310	ccuucucgagccuugagugugc	chr3:52393954..52394014:+
chr16_33381	154.9	303	ccucaggcuguaagugcaggcaga	chr16:10930809..10930854:+
chr2_6770	150.9	295	caguucaaugguguucagcaga	chr2:74539315..74539373:-
chr17_36164	137.9	269	agcaccugccgagcacugaga	chr17:77679375..77679430:+
chr8_20691	134.5	261	acaccgggggguuagagcuucaacc	chr8:115534466..115534535:-
chr1_795	129.9	252	agccucccagucuggccugagu	chr1:55318691..55318744:+
chr8_19921	127.9	243	auagaggauuaaagugaggacc	chr8:125952074..125952134:+

chr8_20229	127.4	250	acagcuucucuauguguggauu	chr8:29067471..29067531:-
chr1_397	126.3	250	uaauguaguugccacuaggaga	chr1:19910421..19910513:+
chr8_20834	122.3	232	cauggcacuggaguagagcau	chr8:140171768..140171839:-
chr12_28907	121.2	236	accgaggggucuuccaggaacuc	chr12:98515838..98515896:-
chr9_22165	119.9	233	aaagaacaaagaaccugagug	chr9:62662721..62662795:-
chr9_21242	119.9	233	aaagaacaaagaaccugagug	chr9:39679635..39679709:+
chr9_22187	119.9	233	aaagaacaaagaaccugagug	chr9:66697252..66697326:-
chr9_21264	119.9	233	aaagaacaaagaaccugagug	chr9:42435995..42436069:+
chr10_24195	114.2	221	caugguccauuuugcucugcuu	chr10:74128708..74128773:-
chr12_28030	112.5	218	cccgcucucccgaagcuguucg	chr12:110468755..110468809:+
chrX_45220	112.2	218	uugcuaacccugccuucccaca	chrX:153944156..153944216:-
chr2_6963	110.2	214	uguguccagcaggagcccggca	chr2:106095631..106095691:-
chr6_16010	108.8	211	ugaucccuuauguucuccuaga	chr6:24818808..24818865:-
chr6_14531	108.8	211	ugaucccuuauguucuccuaga	chr6:24818810..24818867:+
chrX_44303	106.3	206	acuccucauuuguaaacucagg	chrX:129790440..129790500:+
chr1_3740	105.4	205	uagagcuccgaucccauccacc	chr1:149928391..149928448:-
chr13_29328	101.7	197	ccggcugcggcucccaccuugg	chr13:36819432..36819484:+
chr13_29310	99.9	195	ugccuguuuggggaagcugguc	chr13:29936100..29936158:+
chr12_27646	97.3	191	uucaugucccgaacucuccagg	chr12:55952013..55952099:+
chr2_7690	94.3	183	uuuauuucaaaggacagcuggaa	chr2:229330721..229330777:-
chrX_43884	91.7	179	uggggugugcucagagcaggggg	chrX:47388647..47388706:+
chr12_28232	91	177	ucucggggaaugcagagcccacu	chr12:562955..563012:-
chr12_29164	90.9	176	cccccuuucccugagccugcau	chr12:124437441..124437502:-
chr5_14191	86.6	169	uucuuggaccuugcuucagacc	chr5:172133956..172134016:-
chr8_20177	86.4	168	auccuccugaucccuu	chr8:22718265..22718321:-
chr7_18040	86	167	ucuuuuccucaccucguauug	chr7:135996149..135996206:+
chr4_10248	85.3	167	aacugggcauguuggaacuaagcu	chr4:7085386..7085446:+
chr14_30697	84.5	163	uacugggauauuuggagcuucuc	chr14:91859013..91859075:+
chr6_15093	82.2	161	uuggucuucucugcccuccagc	chr6:41223851..41223913:+
chr19_38616	80.9	156	cggggaaagggccgggaagggc	chr19:5978304..5978361:+
chr22_42672	80	155	ugugggcgacaagguuugcugcu	chr22:24437953..24438015:+
chr17_37404	79.1	153	ucugaccacugcauccccuuccc	chr17:74522903..74522966:-
chr7_18697	78.4	158	aaagggauccaaggauugaau	chr7:72937311..72937369:-
chr7_18709	77.7	158	aaagggauccaaggauugaau	chr7:73291885..73291943:-
chr9_22352	76.3	147	cggggcacucgggucuuugcugg	chr9:93566815..93566877:-
chr13_29857	73.5	143	ugguagaaauccagaaucugagg	chr13:49978702..49978769:-
chr8_20936	72.6	140	caguccugagcuccaccccuca	chr8:144099206..144099264:-
chr17_35063	72	132	ugacccuggcucuucucuccagg	chr17:7350068..7350127:+
chr11_25119	70.2	134	uggcccaagaccccagaccaca	chr11:64341847..64341909:+
chr22_43165	69.6	135	ggcuccaccuuccuagguuggc	chr22:26572326..26572384:-
chr14_31162	68.8	132	cucgcguccccgcucccuccucc	chr14:32076763..32076823:-
chr19_40593	68.4	132	ugaggaggaucugaaggauugg	chr19:55225488..55225541:-
chr15_32536	67.4	131	gugcacgugaggauggcucuga	chr15:25705932..25705987:-
chr2_4998	66.9	130	ccagccucagucucuguauccu	chr2:64763719..64763779:+
chr12_28958	66.9	128	uaggucacugggggucagagc	chr12:107747326..107747375:-
chr11_25115	65.6	120	ugcagguucggcuccug	chr11:64320797..64320876:+
chr17_35812	63.8	123	cccgugccuguccccuccucucu	chr17:45411075..45411129:+
chr3_8140	63.8	123	uagccuccaugaauucugauga	chr3:48316381..48316444:+
chrEBV_46455	63.7	123	cggggaucggacuagccuuag	chrEBV:140032..140092:+
chr20_41669	63.6	123	ucuguccugggacgcuuggugucc	chr20:48721226..48721289:-
chr6_14547	63.5	127	uauugaaaggcuccuggggacacc	chr6:26046042..26046110:+
chr19_40002	63.2	122	cucacugcagccucucuuuuccag	chr19:12930527..12930603:-
chrX_44774	61.9	119	auuuaggccuguauaaaucaugg	chrX:66067748..66067804:-
chr3_8690	61.7	130	cuauucaaguucugauc	chr3:148827590..148827628:+
chr11_26062	61.2	118	uagacaaucuguguagagugcu	chr11:18004708..18004770:-
chr10_24112	59.2	113	caucugauggggaauggccugc	chr10:70322916..70322977:-
chr17_36312	59.1	114	uggggccagcuuuggaagccugca	chr17:443795..443860:-
chr19_39495	57.5	113	uagacacagaaucuggaccaca	chr19:51391445..51391503:+
chr10_23129	57.1	111	ugucuguucccugucucucuag	chr10:48966434..48966525:+
chr11_24861	56.9	109	uugcuguuaagauaugggaugg	chr11:33177558..33177627:+
chr22_43204	56.2	109	ccuuugccucuggcucacccucu	chr22:30341887..30341957:-
chrX_44401	55	106	aagcucccccgccccagaga	chrX:153348250..153348308:+
chr1_266	53	105	uccuggcuugugcuucugcaa	chr1:13781849..13781898:+

chr11_26237	51.8	100	caaaaauaggaaguccaaggac	chr11:58504616..58504674:-
chr9_22017	5.9	440	aaaagcuguccacuguagagu	chr9:32456302..32456370:-
chr12_28599	5.7	104	uuguugaacuagggcaggau	chr12:52590997..52591061:-
chrX_44422	5.6	139	cgagggacuccuggccaguguuc	chrX:154346173..154346225:+
chrX_45295	5.6	139	cgagggacuccuggccaguguuc	chrX:154385888..154385940:-
chr22_42747	5.4	269	acccuguccuccaggagcuc	chr22:32128287..32128368:+
chr20_41163	5.2	155	ccucuuccuccagccucugaau	chr20:58449125..58449184:+
chr5_14237	5.2	113771	auccugccgacuaugcca	chr5:175363686..175363761:-
chr14_30165	5.1	403	ucugagcccuguucucccuagg	chr14:20992042..20992092:+
chr2_4858	5.1	120	ugaggggcagagcgaga	chr2:39494998..39495039:+
chr5_13836	5	914	uugacugaagcugauga	chr5:134045214..134045293:-
chr17_35571	5	2557	ucccuguccuccaggagcu	chr17:33228834..33228904:+
chr20_40650	4.9	132	uccugcuggaaagaaccugaga	chr20:299504..299564:+
chr8_20295	4.9	381	ucaggcucaguccccuccau	chr8:41201559..41201634:-
chr14_31014	4.4	120	uucuggaauacugugugagggaa	chr14:18542511..18542587:-

Table S2. The number of significant differentially expressed (SDE) miRNAs between each group of patients, estimated by three methods of analysis (DESeq, edgeR, and NOISeq).

Comparison	common SDE miRNAs	DESeq	edgeR	NOISeq
Healthy subjects vs. spontaneous clarifiers	95	122	128	125
Healthy subjects vs. chronic patients	23	71	83	33
Spontaneous clarifiers vs. chronic patients	0	3	10	1

Table S3. Significant differentially expressed (SDE) miRNAs between HCV spontaneous clarifier subjects and healthy donors. Each column corresponds to one statistical method of analysis: edgeR, DESeq, and NOIseq. Log2FC indicates the fold change of clarifiers vs. donors. The correction for multiple testing was performed with the Benjamini–Hochberg procedure on all three statistical methods. The output of edgeR shows us the FDR (false discovery rate); DESeq shows the padj, which is the p-value adjusted for multiple testing; and NOIseq shows the FDR, which corresponds to the probability of differential expression. NSDE (non-significant differentially expressed). The SDE common miRNAs to all methods are shown in bold.

	**edgeR**		**DESeq**		**NOISeq**	
**miRNA id**	**Log2FC**	**FDR**	**Log2FC**	**FDR**	**Log2FC**	**FDR**
hsa-miR-205-5p	−3.57	5.99 E−04	NSDE	NSDE	−2.87	3.50 E−02
**hsa-miR-4787-5p**	−2.98	8.35 E−11	−3.40	4.69 E−04	−2.76	4.00 E−03
**hsa-miR-7641**	−2.74	1.13 E−22	−2.87	1.34 E−08	−2.84	0.00 E+00
**hsa-miR-4508**	−2.65	1.33 E−25	−2.89	5.45 E−06	−2.80	0.00 E+00
**hsa-miR-122-5p**	−2.63	5.77 E−19	−2.74	1.05 E−12	−2.66	9.00 E−03
**hsa-miR-4497**	−2.62	2.03 E−07	−3.12	1.86 E−02	−2.22	8.00 E−03
**hsa-miR-1248**	−2.31	3.50 E−16	−2.49	1.54 E−04	−2.45	5.00 E−03
**hsa-miR-921**	−2.22	1.78 E−08	−2.81	4.97 E−04	−2.17	1.00 E−02
hsa-miR-3195	−2.09	3.43 E−06	NSDE	NSDE	−2.18	2.20 E−02
**hsa-miR-1229-3p**	−2.00	2.79 E−13	−2.24	7.78 E−07	−2.10	1.00 E−03
**hsa-miR-6819-3p**	−1.91	1.15 E−04	−2.33	2.22 E−03	−1.47	2.30 E−02
**hsa-miR-3196**	−1.89	1.65 E−08	−2.02	6.68 E−03	−1.94	1.40 E−02
hsa-miR-3648	−1.86	6.41 E−05	NSDE	NSDE	−1.86	2.40 E−02
**hsa-chr2_4858**	−1.84	1.29 E−07	−2.12	9.07 E−07	−1.59	2.20 E−02
**hsa-miR-125b-5p**	−1.84	4.73 E−21	−1.88	2.84 E−14	−1.85	0.00 E+00
**hsa-miR-3960**	−1.80	4.21 E−12	−1.99	1.24 E−02	−1.90	7.00 E−03
**hsa-miR-6731-3p**	−1.75	7.70 E−06	−2.25	2.37 E−03	−1.69	2.10 E−02
**hsa-miR-4433b-3p**	−1.72	2.49 E−05	−1.81	2.76 E−02	−1.68	2.10 E−02
**hsa-chr16_34569**	−1.67	1.22 E−12	−1.98	1.99 E−07	−1.84	1.00 E−02
hsa-miR-4792	−1.65	2.56 E−08	NSDE	NSDE	−1.72	1.00 E−02
**hsa-miR-210-5p**	−1.64	9.16 E−13	−1.90	2.15 E−04	−1.79	8.00 E−03
**hsa-chr8_20936**	−1.63	1.79 E−04	−2.30	1.04 E−02	−1.48	2.30 E−02
**hsa-miR-1246**	−1.63	8.82 E−09	−1.83	2.49 E−02	−1.78	2.10 E−02
**hsa-miR-125a-5p**	−1.57	3.13 E−22	−1.65	5.93 E−13	−1.61	0.00 E+00
**hsa-miR-1233-3p**	−1.54	9.09 E−05	−1.97	1.79 E−03	−1.36	2.30 E−02
hsa-miR-3656	−1.53	3.30 E−07	NSDE	NSDE	−1.61	2.50 E−02
**hsa-miR-23a-5p**	−1.50	1.52 E−10	−1.63	2.87 E−03	−1.58	1.00 E−03
**hsa-miR-3609**	−1.45	2.95 E−10	−1.58	5.60 E−05	−1.51	1.00 E−02

**hsa-miR-4433b-5p**	−1.45	1.37 E−07	−1.58	1.17 E−02	−1.50	0.00 E+00
**hsa-miR-6769b-3p**	−1.44	2.09 E−08	−1.65	5.30 E−06	−1.53	2.20 E−02
**hsa-miR-296-5p**	−1.44	4.85 E−10	−1.63	9.15 E−03	−1.52	2.20 E−02
**hsa-miR-6803-3p**	−1.42	1.16 E−14	−1.57	1.12 E−08	−1.50	6.00 E−03
hsa-chr3_8690	−1.42	8.37 E−08	NSDE	NSDE	−1.51	4.00 E−03
**hsa-miR-1908-5p**	−1.39	9.39 E−08	−1.52	1.15 E−04	−1.47	9.00 E−03
**hsa-chr12_29164**	−1.36	5.78 E−04	−1.80	1.20 E−02	−1.34	2.40 E−02
**hsa-miR-1260a**	−1.36	6.78 E−20	−1.48	6.24 E−07	−1.42	0.00 E+00
hsa-miR-1228-3p	−1.36	8.91 E−04	NSDE	NSDE	−1.41	2.60 E−02
**hsa-miR-1260b**	−1.34	5.42 E−20	−1.46	4.98 E−07	−1.40	0.00 E+00
**hsa-miR-423-5p**	−1.34	1.66 E−37	−1.44	1.93 E−11	−1.38	0.00 E+00
**hsa-miR-4526**	−1.32	2.11 E−04	−1.42	2.08 E−04	−1.12	3.40 E−02
**hsa-miR-1249-3p**	−1.31	9.14 E−09	−1.45	1.18 E−02	−1.36	1.00 E−03
hsa-miR-548a-5p	−1.29	1.15 E−03	−1.56	1.13 E−03	NSDE	NSDE
hsa-chr2_6060	−1.29	3.75 E−03	NSDE	NSDE	−1.36	4.70 E−02
**hsa-miR-1234-3p**	−1.28	2.96 E−05	−1.60	1.11 E−03	−1.31	2.00 E−02
**hsa-miR-4687-5p**	−1.26	8.00 E−10	−1.47	9.16 E−03	−1.39	2.20 E−02
**hsa-miR-1276**	−1.21	6.58 E−04	−1.37	4.86 E−04	−1.04	3.80 E−02
hsa-miR-4492	−1.21	1.86 E−03	NSDE	NSDE	−1.28	4.60 E−02
hsa-miR-6747-3p	NSDE	NSDE	−1.20	1.00 E−02	NSDE	NSDE
hsa-miR-1273g-3p	−1.20	7.56 E−05	NSDE	NSDE	−1.27	4.50 E−02
**hsa-miR-99a-5p**	−1.18	1.86 E−08	−1.20	3.29 E−06	−1.17	6.00 E−03
hsa-miR-92b-5p	−1.18	1.90 E−03	NSDE	NSDE	−1.18	4.70 E−02
**hsa-miR-485-5p**	−1.17	1.07 E−03	−1.26	4.28 E−02	−1.22	2.40 E−02
**hsa-chrX_43884**	−1.15	7.46 E−05	−1.38	3.22 E−03	−1.31	2.10 E−02
**hsa-miR-150-5p**	−1.15	3.53 E−14	−1.23	8.84 E−15	−1.18	0.00 E+00
**hsa-miR-625-3p**	−1.14	9.08 E−18	−1.22	5.13 E−12	−1.18	5.00 E−03
**hsa-miR-3150a-5p**	−1.13	5.38 E−04	−1.27	1.08 E−03	−1.00	4.70 E−02
**hsa-miR-1343-3p**	−1.12	3.46 E−07	−1.32	1.82 E−03	−1.23	2.40 E−02
**hsa-miR-615-3p**	−1.12	3.22 E−03	−1.28	1.82 E−02	−1.21	3.90 E−02
**hsa-miR-5189-3p**	−1.12	2.25 E−03	−1.24	6.50 E−03	−1.17	2.50 E−02
**hsa-miR-4787-3p**	−1.11	1.32 E−05	−1.40	3.28 E−02	−1.27	2.30 E−02
hsa-chr1_795	−1.11	1.85 E−02	−1.21	1.55 E−02	NSDE	NSDE
**hsa-miR-6735-5p**	−1.09	1.41 E−04	−1.43	2.93 E−03	−1.25	2.40 E−02
**hsa-chr10_23092**	−1.09	6.53 E−05	−1.28	1.30 E−03	−1.15	2.50 E−02
**hsa-miR-1976**	−1.08	4.69 E−03	−1.56	1.86 E−02	−1.20	2.40 E−02
**hsa-miR-2116-3p**	−1.08	9.80 E−07	−1.34	3.62 E−03	−1.27	2.40 E−02
**hsa-chr19_38809**	−1.08	2.69 E−05	−1.29	9.20 E−05	−1.20	2.20 E−02
**hsa-miR-423-3p**	−1.08	4.69 E−28	−1.17	2.74 E−11	−1.10	0.00 E+00
**hsa-miR-5090**	−1.07	2.89 E−03	−1.28	1.21 E−02	−1.00	4.80 E−02
**hsa-miR-486-3p**	−1.06	1.83 E−06	−1.14	5.12 E−04	−1.09	9.00 E−03
**hsa-miR-211-5p**	−1.05	5.97 E−03	−1.31	2.11 E−02	NSDE	NSDE
hsa-miR-3611	−1.05	8.47 E−03	−1.26	4.28 E−02	NSDE	NSDE
hsa-chr14_31162	−1.05	1.89 E−02	NSDE	NSDE	−1.12	4.60 E−02
**hsa-miR-6734-5p**	−1.04	2.39 E−06	−1.24	5.03 E−06	−1.19	2.50 E−02
**hsa-miR-7704**	−1.04	2.95 E−10	−1.16	1.52 E−03	−1.09	7.00 E−03
**hsa-miR-6511b-3p**	−1.03	3.91 E−08	−1.23	4.17 E−03	−1.14	1.90 E−02
**hsa-miR-328-3p**	−1.03	6.13 E−20	−1.13	1.17 E−10	−1.07	0.00 E+00
hsa-chr20_41163	NSDE	NSDE	NSDE	NSDE	−1.06	4.60 E−02
**hsa-miR-361-3p**	−1.02	1.50 E−17	−1.10	2.96 E−14	−1.05	0.00 E+00
**hsa-miR-125b-2-3p**	−1.02	3.41 E−05	−1.06	1.48 E−03	−1.01	3.50 E−02
hsa-miR-6766-3p	−1.02	9.80 E−03	NSDE	NSDE	−1.07	4.70 E−02
hsa-miR-6741-3p	NSDE	NSDE	−1.11	4.29 E−04	−1.02	2.60 E−02
hsa-miR-1908-3p	NSDE	NSDE	−1.11	1.50 E−02	−1.01	4.70 E−02
hsa-miR-766-3p	NSDE	NSDE	−1.10	2.32 E−05	−1.04	9.00 E−03
hsa-chr9_21973	NSDE	NSDE	−1.10	8.32 E−04	−1.03	2.40 E−02
hsa-miR-1306-5p	NSDE	NSDE	−1.08	2.71 E−04	−1.02	1.90 E−02
hsa-miR-5010-5p	NSDE	NSDE	−1.07	1.18 E−02	NSDE	NSDE
hsa-miR-1292-5p	NSDE	NSDE	−1.07	6.50 E−03	NSDE	NSDE
hsa-miR-4800-3p	NSDE	NSDE	−1.06	4.00 E−02	NSDE	NSDE
hsa-chr10_24527	NSDE	NSDE	−1.06	3.28 E−03	NSDE	NSDE
hsa-miR-25-5p	NSDE	NSDE	−1.05	1.34 E−08	−1.02	2.20 E−02
hsa-miR-4742-5p	NSDE	NSDE	−1.05	1.21 E−02	NSDE	NSDE
hsa-miR-4796-3p	NSDE	NSDE	−1.05	6.28 E−05	NSDE	NSDE

hsa-miR-193b-3p	NSDE	NSDE	−1.05	8.86 E−08	NSDE	NSDE
hsa-miR-150-3p	NSDE	NSDE	−1.04	3.21 E−08	NSDE	NSDE
hsa-miR-3130-3p	NSDE	NSDE	−1.03	1.36 E−02	NSDE	NSDE
hsa-miR-4536-3p	NSDE	NSDE	1.14	5.44 E−03	NSDE	NSDE
hsa-miR-4728-3p	NSDE	NSDE	NSDE	NSDE	−1.02	4.90 E−02
hsa-miR-139-3p	−1.01	1.69 E−02	NSDE	NSDE	−1.03	5.00 E−02
hsa-miR-5091	1.01	1.44 E−02	NSDE	NSDE	NSDE	NSDE
hsa-miR-193a-3p	1.03	8.57 E−07	NSDE	NSDE	1.01	2.80 E−02
hsa-miR-223-5p	1.04	1.13 E−18	NSDE	NSDE	1.01	0.00 E+00
**hsa-miR-26a-2-3p**	1.05	2.49 E−09	1.00	4.25 E−06	1.01	3.30 E−02
hsa-miR-223-3p	1.06	2.20 E−10	NDE	NSDE	1.00	0.00 E+00
**hsa-miR-18a-5p**	1.07	2.32 E−15	1.03	2.13 E−11	1.06	4.00 E−03
**hsa-miR-660-5p**	1.08	1.27 E−21	1.04	6.35 E−25	1.06	0.00 E+00
hsa-miR-581	1.08	3.60 E−03	1.46	4.93 E−02	NSDE	NSDE
**hsa-miR-340-5p**	1.09	9.08 E−18	1.04	1.80 E−18	1.06	0.00 E+00
**hsa-miR-190b**	1.09	3.51 E−15	1.04	6.14 E−15	1.07	1.20 E−02
hsa-miR-5096	1.09	3.06 E−07	NSDE	NSDE	1.01	4.60 E−02
**hsa-miR-3613-5p**	1.10	6.52 E−12	1.04	3.31 E−08	1.05	1.20 E−02
hsa-miR-15b-3p	1.10	5.14 E−04	NSDE	NSDE	1.11	2.90 E−02
hsa-miR-939-5p	NSDE	NSDE	−1.18	1.44 E−04	−1.09	2.40 E−02
**hsa-miR-4461**	1.11	2.60 E−04	1.19	1.72 E−02	1.16	4.00 E−02
**hsa-chr1_2076**	1.15	7.59 E−08	1.07	1.27 E−11	1.11	1.20 E−02
hsa-chr17_37404	1.17	2.50 E−03	NSDE	NSDE	NSDE	NSDE
**hsa-miR-708-5p**	1.19	3.81 E−03	1.16	1.06 E−02	1.21	3.40 E−02
**hsa-miR-449c-5p**	1.20	5.12 E−04	1.13	1.43 E−03	1.16	4.90 E−02
**hsa-miR-619-5p**	1.21	1.46 E−08	1.11	1.13 E−03	1.15	3.40 E−02
**hsa-miR-708-3p**	1.21	3.24 E−03	1.17	1.54 E−03	1.19	4.90 E−02
**hsa-miR-655-3p**	1.25	6.51 E−04	1.27	2.07 E−03	1.14	5.00 E−02
hsa-miR-219b-3p	1.26	4.93 E−04	1.30	6.44 E−04	NSDE	NSDE
hsa-miR-5095	1.28	8.78 E−05	1.15	6.70 E−03	NSDE	NSDE
**hsa-miR-1273d**	1.29	1.09 E−07	1.19	2.67 E−04	1.21	3.30 E−02
hsa-miR-378b	1.30	6.00 E−04	1.31	7.93 E−04	NSDE	NSDE
**hsa-chr1_1467**	1.32	3.38 E−08	1.26	2.85 E−07	1.29	2.60 E−02
**hsa-miR-9-5p**	1.33	6.64 E−15	1.29	1.65 E−09	1.29	1.00 E−03
**hsa-miR-582-5p**	1.38	6.04 E−13	1.34	2.12 E−08	1.36	0.00 E+00
**hsa-miR-21-3p**	1.42	1.63 E−21	1.37	5.03 E−27	1.41	0.00 E+00
**hsa-miR-1537-5p**	1.44	1.91 E−06	1.68	4.25 E−06	1.32	3.30 E−02
**hsa-miR-362-5p**	1.45	1.06 E−14	1.39	1.26 E−05	1.40	5.00 E−03
**hsa-miR-5585-3p**	1.49	8.00 E−10	1.39	1.01 E−04	1.41	3.30 E−02
**hsa-miR-944**	1.49	2.12 E−13	1.44	8.46 E−14	1.45	1.10 E−02
hsa-miR-6753-3p	1.51	2.00 E−04	1.28	7.64 E−04	NSDE	NSDE
**hsa-chr5_13836**	1.51	2.36 E−07	1.39	1.70 E−08	1.41	2.80 E−02
**hsa-miR-382-3p**	1.55	1.42 E−05	1.57	6.50 E−03	1.60	1.80 E−02
**hsa-miR-4531**	1.56	5.59 E−13	1.51	1.70 E−13	1.52	7.00 E−03
**hsa-miR-582-3p**	1.59	4.43 E−16	1.53	2.72 E−15	1.56	2.00 E−03
**hsa-miR-6723-5p**	1.72	7.50 E−06	1.96	2.66 E−04	1.40	2.90 E−02
**hsa-miR-4424**	1.78	6.57 E−06	1.72	1.05 E−02	1.62	2.40 E−02
**hsa-miR-4284**	2.27	1.89 E−10	2.26	2.71 E−04	2.25	0.00 E+00
**hsa-miR-4485-3p**	2.38	2.33 E−09	2.33	4.28 E−02	2.32	0.00 E+00
hsa-chrX_44362	2.43	2.44 E−07	NSDE	NSDE	2.38	1.10 E−02
**hsa-miR-1973**	2.44	4.87 E−08	2.46	1.49 E−03	2.38	1.20 E−02
hsa-miR-124-5p	2.61	8.08 E−04	NSDE	NSDE	NSDE	NSDE
**hsa-miR-1296-3p**	2.89	1.21 E−10	2.92	3.64 E−04	2.82	4.00 E−03
hsa-miR-1299	3.12	1.41 E−05	NSDE	NSDE	3.01	1.90 E−02
**hsa-chr18_37981**	3.34	3.24 E−11	3.50	1.11 E−03	3.00	4.00 E−03
**hsa-miR-124-3p**	3.77	1.62 E−07	4.23	2.07 E−03	3.22	4.00 E−03

Table S4. Significant differentially expressed (SDE) miRNAs between HCV+ chronic patients and healthy donors. Each column corresponds to one statistical method of analysis: edgeR, DESeq, and NOIseq. Log2FC indicates the fold change of chronic vs. donors. The correction for multiple testing was performed with the Benjamini–Hochberg procedure on all three statistical methods. The output of edgeR shows us the FDR (false discovery rate); DESeq shows the padj, which is the p-value adjusted for multiple testing; and NOIseq shows the FDR, which corresponds to the probability of a differential expression. NSDE (non-significant differentially expressed). The SDE common miRNAs to all methods are shown in bold.

	**edgeR**		**DESeq**		**NOIseq**	
**miRNA id**	**logFC**	**FDR**	**logFC**	**padj**	**log2FC**	**FDR**
hsa-miR-205-5p	−4.43	2.42 E−05	NSDE	NSDE	NSDE	NSDE
**hsa-miR-4787-5p**	−2.65	3.94 E−09	−2.91	2.94 E−03	−2.44	1.15 E−02
**hsa-miR-3648**	−2.41	4.11 E−07	−2.82	4.58 E−02	−2.15	4.59 E−02
hsa-miR-4497	−2.31	2.65 E−06	NSDE	NSDE	−2.10	4.49 E−02
**hsa-miR-4508**	−2.2	3.88 E−19	−2.39	4.11 E−04	−2.25	1.09 E−02
hsa-chr2_6060	−2.1	2.65 E−06	NSDE	NSDE	−2.09	4.99 E−02
**hsa-miR-122-5p**	−1.92	4.33 E−11	−1.92	7.03 E−08	−1.81	2.75 E−02
**hsa-miR-3960**	−1.85	3.65 E−12	−2.02	1.88 E−02	−1.87	1.19 E−02
hsa-miR-4532	−1.85	1.10 E−06	−2.02	8.74 E−03	NSDE	NSDE
**hsa-miR-3196**	−1.85	4.06 E−08	−1.96	1.86 E−02	−1.84	3.98 E−02
hsa-miR-1273g-3p	−1.8	5.06 E−09	NSDE	NSDE	−1.79	4.18 E−02
hsa-chr3_8690	−1.74	1.40 E−10	NSDE	NSDE	−1.71	9.93 E−03
**hsa-miR-1248**	−1.74	7.96 E−10	−1.86	1.00 E−02	−1.74	3.73 E−02
hsa-miR-4492	−1.69	1.24 E−05	NSDE	NSDE	−1.70	4.19 E−02
hsa-miR-3195	−1.67	2.10 E−04	NSDE	NSDE	NSDE	NSDE
**hsa-miR-1229-3p**	−1.65	1.06 E−09	−1.81	9.09 E−05	−1.69	2.86 E−02
**hsa-miR-23a-5p**	−1.54	1.40 E−10	−1.63	2.87 E−03	−1.52	1.67 E−05
hsa-miR-3687	−1.54	1.15 E−05	NSDE	NSDE	NSDE	NSDE
**hsa-miR-7641**	−1.49	7.60 E−08	−1.58	6.34 E−03	−1.48	1.40 E−02
hsa-miR-1246	−1.48	2.50 E−07	NSDE	NSDE	−1.49	4.31 E−02
hsa-miR-4792	−1.48	8.94 E−07	NSDE	NSDE	−1.44	4.13 E−02
**hsa-miR-125b-5p**	−1.43	8.55 E−13	−1.46	1.06 E−08	−1.36	1.00 E−04
hsa-miR-3656	−1.4	3.90 E−06	NSDE	NSDE	NSDE	NSDE
**hsa-miR-1908-3p**	−1.38	9.40 E−06	−1.56	1.73 E−03	−1.43	4.13 E−02
hsa-miR-4516	−1.36	2.60 E−05	NSDE	NSDE	NSDE	NSDE
hsa-miR-6819-3p	−1.35	6.13 E−03	−1.68	4.96 E−02	NSDE	NSDE
hsa-miR-4796-3p	−1.33	7.98 E−07	−1.48	2.58 E−07	NSDE	NSDE
hsa-chr17_36164	−1.32	7.99 E−03	−1.50	1.98 E−02	NSDE	NSDE
hsa-chr8_20936	−1.32	2.07 E−03	NSDE	NSDE	NSDE	NSDE
**hsa-miR-1249-3p**	−1.3	1.97 E−08	−1.43	2.18 E−02	−1.27	8.91 E−03
**hsa-miR-210-5p**	−1.28	2.06 E−08	−1.48	1.01 E−02	−1.33	4.22 E−02
hsa-miR-1233-3p	−1.28	9.73 E−04	−1.67	1.66 E−02	NSDE	NSDE
hsa-miR-296-5p	−1.26	7.64 E−08	−1.43	4.58 E−02	NSDE	NSDE
**hsa-miR-4687-5p**	−1.25	1.50 E−09	−1.44	1.80 E−02	−1.28	4.23 E−02
**hsa-miR-2116-3p**	−1.25	1.97 E−08	−1.50	1.47 E−03	−1.36	4.33 E−02
hsa-miR-4433b-5p	−1.24	1.16 E−05	NSDE	NSDE	−1.24	1.07 E−02
hsa-miR-3917	−1.24	2.86 E−03	−1.52	4.56 E−03	NSDE	NSDE
hsa-miR-615-3p	−1.2	1.98 E−03	−1.33	2.15 E−02	NSDE	NSDE
**hsa-chr16_34569**	−1.19	1.29 E−07	−1.40	4.11 E−04	−1.25	4.43 E−02
hsa-chr2_6327	−1.13	3.08 E−03	−1.17	1.16 E−02	NSDE	NSDE
hsa-miR-1228-3p	−1.12	8.12 E−03	NSDE	NSDE	NSDE	NSDE
hsa-miR-3611	−1.1	6.13 E−03	−1.26	4.50 E−02	NSDE	NSDE
**hsa-miR-125a-5p**	−1.09	7.26 E−11	−1.14	2.86 E−06	−1.04	0.00 E+00
hsa-chr12_29164	−1.08	6.37 E−03	NSDE	NSDE	NSDE	NSDE
hsa-miR-3607-5p	−1.08	8.29 E−03	−1.40	4.83 E−02	NSDE	NSDE
hsa-miR-921	−1.06	5.18 E−03	NSDE	NSDE	NSDE	NSDE
hsa-miR-6735-5p	NSDE	NSDE	−1.27	1.24 E−02	NSDE	NSDE
hsa-miR-548a-5p	NSDE	NSDE	−1.24	1.86 E−02	NSDE	NSDE
hsa-miR-6741-3p	−1.06	3.35 E−05	−1.19	3.15 E−04	NSDE	NSDE
hsa-miR-133a-3p	−1.04	1.37 E−03	−1.06	7.42 E−03	NSDE	NSDE
hsa-miR-758-5p	−1.04	4.57 E−02	NSDE	NSDE	NSDE	NSDE
hsa-miR-3607-3p	−1.03	1.83 E−06	−1.14	3.75 E−02	NSDE	NSDE

hsa-miR-1234-3p	NSDE	NSDE	−1.13	2.42 E−02	NSDE	NSDE
hsa-miR-1908-5p	NSDE	NSDE	−1.11	2.08 E−02	NSDE	NSDE
hsa-miR-935	−1.02	8.96 E−03	NSDE	NSDE	NSDE	NSDE
hsa-miR-4485-5p	1.01	1.96 E−02	NSDE	NSDE	NSDE	NSDE
hsa-miR-2467-5p	1.04	2.80 E−08	1.08	1.49 E−07	NSDE	NSDE
hsa-miR-6813-5p	NSDE	NSDE	−1.08	2.80 E−04	NSDE	NSDE
hsa-miR-1306-5p	NSDE	NSDE	−1.06	8.21 E−04	NSDE	NSDE
hsa-miR-423-5p	NSDE	NSDE	−1.02	2.94 E−05	NSDE	NSDE
hsa-miR-6747-3p	NSDE	NSDE	−1.02	3.16 E−02	NSDE	NSDE
hsa-miR-1260a	NSDE	NSDE	−1.01	3.64 E−03	NSDE	NSDE
hsa-miR-1343-3p	NSDE	NSDE	−1.01	4.49 E−02	NSDE	NSDE
hsa-miR-1260b	NSDE	NSDE	−1.00	3.25 E−03	NSDE	NSDE
hsa-miR-423-3p	NSDE	NSDE	−1.00	7.03 E−08	NSDE	NSDE
hsa-miR-129-1-3p	NSDE	NSDE	1.02	3.52 E−02	NSDE	NSDE
hsa-miR-944	1.05	8.94 E−07	1.02	3.67 E−07	NSDE	NSDE
hsa-miR-3614-3p	1.06	6.17 E−04	1.26	3.74 E−03	NSDE	NSDE
hsa-miR-378h	1.06	1.01 E−03	1.06	1.32 E−03	NSDE	NSDE
**hsa-miR-582-3p**	1.07	1.06 E−07	1.04	1.54 E−07	1.15	2.12 E−02
hsa-miR-449a	NSDE	NSDE	1.05	3.39 E−02	NSDE	NSDE
hsa-miR-200a-5p	NSDE	NSDE	1.06	4.65 E−02	NSDE	NSDE
hsa-miR-451a	1.08	1.70 E−02	NSDE	NSDE	NSDE	NSDE
hsa-chr4_10248	1.09	2.64 E−03	1.12	3.94 E−03	NSDE	NSDE
hsa-miR-3614-5p	1.11	4.06 E−08	1.06	6.58 E−05	NSDE	NSDE
hsa-miR-1537-5p	1.12	5.41 E−04	1.31	8.13 E−04	NSDE	NSDE
hsa-miR-4531	1.18	1.19 E−07	1.17	1.61 E−07	NSDE	NSDE
hsa-miR-382-3p	1.19	1.50 E−03	NSDE	NSDE	NSDE	NSDE
hsa-miR-6753-3p	1.19	6.91 E−03	1.16	1.86 E−02	NSDE	NSDE
hsa-miR-129-5p	1.19	4.60 E−03	1.26	1.83 E−02	NSDE	NSDE
hsa-chr1_1467	1.24	3.51 E−07	1.25	3.17 E−06	NSDE	NSDE
hsa-miR-3128	1.25	1.79 E−04	1.64	3.61 E−04	NSDE	NSDE
hsa-miR-183-5p	1.33	3.47 E−03	NSDE	NSDE	NSDE	NSDE
**hsa-miR-21-3p**	1.39	5.97 E−20	1.38	2.37 E−25	1.49	0.00 E+00
hsa-miR-3681-5p	1.4	9.28 E−05	1.26	4.39 E−04	NSDE	NSDE
hsa-miR-412-5p	1.41	1.82 E−03	NSDE	NSDE	NSDE	NSDE
hsa-miR-6723-5p	1.43	3.81 E−04	1.65	3.45 E−03	NSDE	NSDE
hsa-miR-4461	1.49	3.76 E−07	1.65	1.29 E−03	NSDE	NSDE
hsa-chr5_13836	1.68	1.07 E−08	1.61	2.69 E−10	NSDE	NSDE
hsa-chr18_37981	1.73	1.28 E−03	NSDE	NSDE	NSDE	NSDE
hsa-miR-4424	1.79	7.91 E−06	1.80	1.92 E−02	NSDE	NSDE
hsa-miR-1973	2.36	2.05 E−07	2.44	3.97 E−03	NSDE	NSDE
**hsa-miR-4284**	2.4	7.26 E−11	2.43	1.53 E−04	2.49	1.57 E−02
hsa-miR-4485-3p	2.62	1.70 E−10	NSDE	NSDE	2.69	5.50 E−03
**hsa-miR-1296-3p**	2.87	3.68 E−10	2.96	5.76 E−04	2.92	1.56 E−02
hsa-chrX_44362	2.98	7.93 E−10	NSDE	NSDE	3.07	3.17 E−02
hsa-miR-124-5p	4.33	4.96 E−07	5.19	2.13 E−02	NSDE	NSDE
**hsa-miR-124-3p**	4.75	3.68 E−10	5.26	1.50 E−04	4.33	2.00 E−15

Table S5. Significant differentially expressed (SDE) miRNAs between HCV+ chronic patients and spontaneous clarifiers subjects. Each column corresponds to one statistical method of analysis: edgeR, DESeq, and NOIseq. Log2FC indicates the fold change of HCV+ chronic patients vs. spontaneous clarifiers. The correction for multiple testing was performed with the Benjamini–Hochberg procedure on all three statistical methods. The output of edgeR shows us the FDR (false discovery rate); DESeq shows the padj, which is the p-value adjusted for multiple testing; and NOIseqBIO shows the FDR, which corresponds to the probability of a differential expression. NSDE (non-significant differentially expressed).

	**edgeR**		**DESeq**		**NOIseq**	
miRNA id	**logFC**	**FDR**	**logFC**	**FDR**	**log2FC**	**FDR**
hsa-miR-7641	1.25	2.92 E−04	NSDE	NSDE	1.36	3.64 E−02
hsa-miR-3917	−1.40	9.34 E−03	−1.61	2.00 E−04	NSDE	NSDE
hsa-chr18_37981	−1.62	1.03 E−02	NSDE	NSDE	NSDE	NSDE
hsa-miR-3687	−1.19	1.03 E−02	NSDE	NSDE	NSDE	NSDE
hsa-miR-2115-5p	−1.28	1.17 E−02	−1.32	3.18 E−04	NSDE	NSDE
hsa-chr2_4858	1.27	1.24 E−02	1.44	4.11 E−04	NSDE	NSDE
hsa-miR-1299	−2.25	1.77 E−02	NSDE	NSDE	NSDE	NSDE
hsa-miR-183-5p	1.27	3.48 E−02	NSDE	NSDE	NSDE	NSDE
hsa-miR-4755-5p	1.01	4.13 E−02	NSDE	NSDE	NSDE	NSDE
hsa-miR-708-5p	−1.11	4.61 E−02	NSDE	NSDE	NSDE	NSDE

Table S6. miRNA validation primers.

**miRNA id**	**miRNA sequence**	**Primer Sequence (Forward)**
hsa-miR-21-3p	CAACACCAGUCGAUGGGCUGU	CAACACCAGTCGATGGGCTGT
hsa-miR-23a-5p	AUCACAUUGCCAGGGAUUUCC	ATCACATTGCCAGGGATTTCC
RNU44		CCTGGATGATGATAGCAAATGC

Table S7. hsa-miR-21-3p and has-miR-23a-5p expression by qRT-PCR in a set of 66 different patients.

	**All samples**	**CHC**	**SC**	**HC**
**No.**	66	22	22	22
**hsa-miR-21-3p**	4.60 (2.24; 7.95)	5.42 (3.72; 10.36)	4.99 (2.75; 8.04)	2.17 (1.07; 5.67)
**hsa-miR-23a-5p**	0.62 (0.38; 1.14)	0.66 (0.48; 1.14)	0.89 (0.47; 1.32)	0.37 (0.30; 0.84)
	**p-value KW**	**SC vs. HC p-value***	**CHC vs. HC p-value***	**CHC vs. SC p-value***
**hsa-miR-21-3p**	**0.005**	**0.017**	**0.003**	0.318
**hsa-miR-23a-5p**	**0.026**	**0.013**	0.054	0.313

Values expressed as absolute numbers median (percentile 25; percentile 75). p-values were estimated by a nonparametric Kruskal–Wallis test and Mann–Whitney. Statistically significant differences are shown in bold.

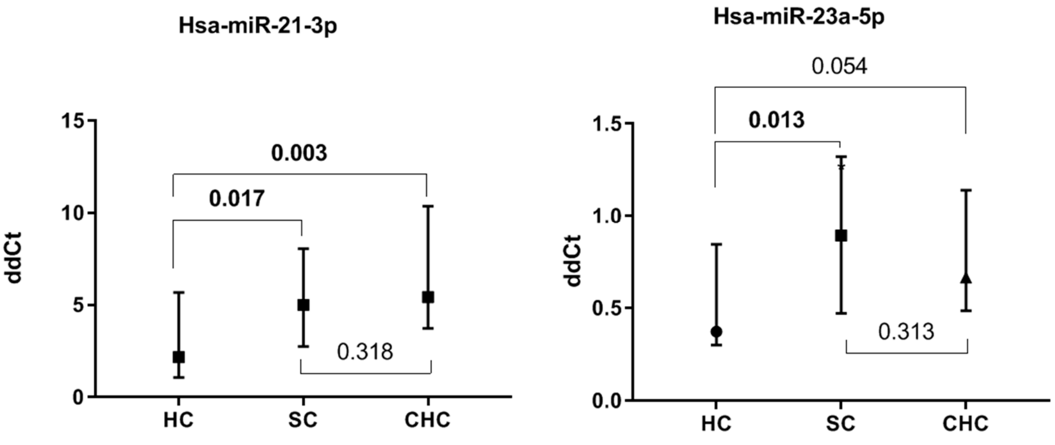

Figure S1. hsa-miR-21-3p and has-miR-23a-5p expression by qRT-PCR in a set of 66 different patients.

Experimental Section

Samples were recruited from Hospital Universitario Virgen de Valme (Seville), Hospital Universitario Marqués de Valdecilla (Santander), and Hospital Universitario Infanta Leonor (Madrid) from 2014 to 2017. All samples were processed at the National Center for Microbiology (Majadahonda). The study protocol conformed to the ethical guidelines of the 1975 Declaration of Helsinki as reflected in a priori approval by the Ethics Committee of Institute of Health Carlos III (CEI PI 11_2015-V4), and written informed consent was obtained from all patients involved.

Patient Groups

Ninety-six Caucasian patients were recruited and classified in three groups: a) HCV spontaneous clarifiers (SC), individuals who spontaneously resolved acute HCV infection (serum positive antibody and negative PCR); b) chronic hepatitis C (CHC) treatment-naïve patients (detectable HCV RNA by PCR); and c) healthy controls (HC) that were never infected with HCV (antibody and PCR negative). The exclusion criteria included the following: pregnancy; individuals below 18 years old; previously HCV treated; advance liver fibrosis; clinical evidence of hepatic decompensation; active drug or alcohol addiction; alcohol-induced liver injury; HBV active infection; anti-HIV antibody; opportunistic infections; and other concomitant diseases such as diabetes, nephropathies, autoimmune disease, hemochromatosis, cryoglobulinemia, primary biliary cirrhosis, Wilson´s disease, α1-antitrypsin deficiency, and neoplasia.

Clinical Records

HCV-related clinical and epidemiological data were obtained from medical records as the year of infection, time since spontaneous clarification, route of transmission, fibrosis stage, HCV viral load, HCV genotype, and genotype of rs12979860 polymorphism at *interferon lambda 4 (gene/pseudogene)* (*IFNL4*). The liver stiffness measurement (LSM) was assessed by transient elastometry (FibroScan®, Echosens, Paris, France) and expressed in kilopascals (kPa) (1). Subjects were stratified according to cut-offs of LSM: <7.1 kPa (F0–F1 absence or mild fibrosis) and 7.1–9.4 kPa (F2 significant fibrosis). The HCV-RNA viral load was measured by quantitative polymerase chain reaction (qPCR) (Cobas Amplicor HCV Monitor Test, Branchburg, NJ, USA; and COBAS AmpliPrep/COBAS TaqMan HCV test). The results were reported in International Units per milliliter (IU/mL), with a lower limit of detection of 10 IU/mL. In the case of patients with a previous history of intravenous drug use (IDU), the year of infection was estimated since the first year they shared needles and other injection paraphernalia (2).

Clinical characteristics of metabolic status were also recorded; the body mass index (BMI) was calculated as the weight in kilograms divided by the square of the height in meters, and the following biochemical parameters were also recorded: glucose level, total cholesterol (TC), low density lipoprotein (LDL), high density lipoprotein (HDL), and triglycerides (TG). Homocysteine has been evaluated as a measure of lipid peroxidation, and values higher than 15 μmol/L were considered high. Different lipid ratios were calculated to assess lipid metabolism deregulation: LDL/HDL, normal values below 2.0; AI, atherogenic index calculated as TC/HDL, with cut-off values of low risk <5% for men and <4.5% for women, moderate risk 5–9% for men and 4.5–7% for women; AIP, atherogenic index for plasma considering AIP > 0.21 as high risk; and LCI, lipoprotein combine index defined as the ratio of TC∗TG∗LDL to HDL-C.

Also, the following biochemical parameters of liver function were recorded: (GOT) glutamate oxaloacetate transaminase; glutamic-pyruvic transaminase (GPT); gamma-glutamyl transferase (GGT); alkaline phosphatase (ALP); and total bilirubin (TB). Two noninvasive scoring systems for the assessment of hepatic fibrosis were also collected: AST to platelet ratio index (APRI) and fibrosis-4 index for liver fibrosis (FIB-4).

Total RNA Isolation and Quality Control

Peripheral venous blood samples were collected in EDTA tubes, and PBMCs were isolated within the first 4 hours after extraction in order to minimize miRNA expression profile. Total RNA including miRNAs were isolated with the miRNeasy Mini kit (Qiagen). RNA was eluted in 30 ul of nuclease-free water, and the RNA concentration was measured by Nanodrop. Quality and integrity were evaluated by the Bioanalyzer 2100 with Agilent RNA 6000 Nano kit (Agilent, catalog no. 5067-1511). Only the samples with a high quality of RNA (RIN > 7.5) were sequenced.

High Throughput Sequencing of Small RNA

Small RNA library synthesis and sequencing were performed at the Centre for Genomic Regulation (CRG) in Barcelona (Spain). Small RNA libraries were constructed with Illumina's TruSeq Small RNA kit v.4 (Illumina, ref. RS-200-0012). In brief, 1 microgram of total RNA was used and 3′ and 5′ adapters were sequentially ligated to the ends of RNA < 200 nt long and reverse transcribed to generate cDNA (SuperScript II, ref. 18064-014, Invitrogen) with a specific primer (RNA RT Primer) complementary to the 3′ RNA adapter. The cDNA was further amplified by PCR (11 cycles) using a common primer complementary to the 3′ adapter and a 5′ primer containing 1 of 48 index sequences. Libraries were size-selected using 6% Novex® TBE Gels (ref. EC6265BOX, Life Technologies). Fragments with insert sizes of 18 to 36 bp were cut from the gel, and DNA was purified, quantified, and pooled for multiplexed sequencing. Final libraries were analyzed using Agilent DNA 1000 chip to estimate the quantity and to check the size distribution and were then quantified by qPCR using the KAPA Library Quantification Kit (ref. KK4835, KapaBiosystems) prior to amplification with Illumina's cBot. Sequencing was performed in the Illumina HiSeq2500, Single Read, 50nts (1x50). Four pools of 24 barcoded samples were prepared, and each pool was sequenced on one line of the same run to limit the batch effect.

MiRNA Validation by Quantitative RT-PCR

Five hundred ng of total RNA was reverse transcribed into complementary DNA with the qScript microRNA cDNA synthesis kit, following the manufacturer instructions. Briefly, miRNAs were polyadenylated, and the poly(A) tailed miRNAs were converted into first-stranded cDNA with an oligo-dT adapter primer. Individual miRNAs were quantified in a SYBR green quantitative PCR reaction by PerfeCta SYBR Green SuperMix (2x) (Quantabio) with a forward primer (sequence of de selected mature miRNA) and the PerfeCta universal PCR primer (specific to the unique sequence of the oligo-dT adapter primer). Sequences of the miRNA nucleotides were extracted from the miRBase Reslease 21 (www.mirbase.org) (1). The PCR efficiency of each miRNA amplicon was evaluated with a standard curve using a 10-fold dilution series of total RNA from a donor buffy. Only miRNA amplicons with an efficiency higher than 1.9 were accepted.

Real time reactions were performed on a Roche LightCycler 480 with a 96-well reaction plate. There is no universal endogenous control for every tissue, and the suitable endogenous control has to be validated for each sample set (2). Endogenous control selection was performed according to the stability of their gene expression in the three groups of study with geNorm(3), BestKeeper (4), and Normfinder (5). Five small nuclear RNA (snRNA) commonly used as endogenous control from miRNA expression studies were tested for suitability: the RNA U1 (RNU1), U2 (RNU2), U6 (RNU6), U44 (RNU44), and the small nucleolar RNA C/D box 48 (SNORD48).

Raw data was exported to Factor qPCR to normalize and remove multiplicative between-run variations of the PCR experiment with multiple plates. (6). Data were analyzed using the $\Delta\Delta$CT method.

Data Processing Pipeline: Bioinformatics Analysis

We set up a specific bioinformatic pipeline for our data, which includes the following steps.

Known miRNA Identification from High-Throughput Sequencing Data

The raw data were initially filtered out for reads with ambiguous base calls, which did not meet the Illumina chastity filter based on quality measures, and the reads were sorted on sample type based on matches to the multiplex tags. Quality control of the remaining sequences was performed by using FastQC (v0.11.3)(3). Adapter sequences as well as low-quality base calls ($q < 20$) were trimmed with cutadapt (v. 1.13). Adapter-trimmed reads were processed with miRDeep2 (v. 0.0.7) (4), which identifies known and novel miRNAs from the dataset. This software maps processed reads to the human reference genome (GRCh38) (mapper.pl module) based on Bowtie1. Only the alignments with 0 mismatches in the seed region and those that do not map to more than five different loci in the genome were retained. Quantification was performed with the quantifier.pl module, which maps the reads to miRNA precursors (obtained from miRBase v20 which contains 1917 precursors and 2654 mature sequences) and determines the expression of the corresponding miRNAs. Only miRNAs with a minimum of total 100 counts in all samples were retained.

De Novo miRNA Identification

The miRDeep2.pl module was used to discover potential novel miRNAs. Briefly, reads were aligned to the human reference genome and candidate pre-miRNA sequences were extracted and score-assigned based on the ability of the precursor to fold to a pre-miRNA-like secondary hairpin structure (4). Mature miRNAs from related species such as *Gorilla gorilla, Pongo pygmaeus, Pan troglodytes*, and *Pan paniscus* were used to predict with a higher confidence the new miRNAs. A miRDeep2 score was assigned to each predicted miRNAs, which represents the probability that the sequence is a true miRNA precursor based on the theory of miRNA processing by dicer as well as the actual data and the alignment pattern of the reads. Only those miRNAs that fulfill the following criteria were selected: (1) A miRDeep2 score cut-off of >4; (2) an estimated probability that the miRNA candidate is a true positive > 0; (3) the total read counts of the predicted mature are >100; and (4) a significant randfold p-value of the excised potential miRNA hairpin (5).

Statistical Analysis of High-Throughput miRNA Data

Multivariate Data Analysis

We used dimensional reduction methods as the Principal Component Analysis (PCA) to visualize whether the experimental samples were clustered according to the groups of patients and to identify unwanted sources of noise. We used the NOISeq PCA function, which allows us to plot the loading values, that is, the projection of the genes on the new principal components or the scores, which are the projections of the samples (observations) on the space created by the new components.

Statistical Analysis for Significant Differentially Expressed miRNAs

Currently, there are not specific software packages designed to normalize miRNA sequencing data; for this reason, three normalization methods commonly used for RNA sequencing analysis were tested.

<u>Normalization methods</u>: (1) reads per kilobase million (RPKM) by DESeq (6) (v 1.28.0); (2) trimmed mean of M-values normalization method (TMM) by edgeR (v 3.18.1) (7); and (3) upper quantile normalization (UPERQ) by NOISeq (v 2.14.1) (8). Each of these methods is described briefly: (1) *DESeq* uses a negative binomial distribution of the count data to perform a differential expression analysis and incorporates data-driven prior distributions to estimate the dispersion and fold changes (6). (2) *TMM* normalization takes into account the composition of the RNA population being sampled, which is neglected in total count scaling, and gives us the proportion of counts for a specific target across all samples. *edgeR fit a negative binomial generalized log-linear model to read counts for each gene.* Both methods are parametric, we also included a nonparametric method in the comparison: (3) Quantile normalization is nonscaling and assumes that the overall distribution of signal intensity does not change. NOISeq for biological replicates (NOISeqBIO) corrects data by length and implements an empirical Bayes approach that improves the handling of biological variability specific to each gene.

Significantly differentially expressed (SDE) miRNAs were calculated by a fold change (FC) of >2 and a statistically significant t test p-value < 0.05 adjusted by the false discovery rate (FDR) using the Benjamin–Hochberg correction. The testing for differentially expressed genes was performed as follow: (1) DESeq fit a generalized linear model for each miRNA with the fold change estimate shrunken by empirical Bayes; the function *nbionmTest* was used to estimate differences between groups. (2) edgeR used a likelihood ratio test for determining the differential expression among groups of patients, and it is performed with the *glmFit()* and *glmLRT()* functions. (3) NOISeq is a nonparametric method that improves the control of the high FDR in experiments with biological replicates, inspired by the work of Efron et al. (9). Low-count filtering was performed by the Wilcoxon test method, which is recommended for our number of samples. The probability of differential expression is equivalent to 1-FDR, where FDR is considered an adjusted p-value (0.05).

Venn Diagram Analysis and Clustering

A Venn Diagram was performed to determine the overlapping SDE miRNAs, which were defined as common SDE miRNAs in the three method of normalization and statistical analysis. The overlapping list of each group of comparison was determined as the SDE miRNAs for subsequent analysis.

An unsupervised hierarchical clustering of differentially expressed genes between patient groups was performed using hclust algorithm (heatmap3, R package) with the average linkage rule (UPGMA method).

miRNA-Based Target Prediction and Pathway Enrichment Analysis of the Target Genes

The web-based computational tool DIANA-miRPath v3.0 (10) was used for the in silico target identification of the SDE miRNAs. The analysis was based on experimentally supported targets predicted in silico and annotated in DIANA-TarBase v.8.0 (11). This tool also performs a pathway union analysis of those miRNAs targets, which is performed for Kyoto Encyclopedia of Genes and Genomes (KEGG) pathways (12). Enrichment p-values (Fischer's exact test with hypergeometric distribution) were corrected for false discovery rate (FDR) and were considered significant when adjusted for $p \leq 0.05$.

Next, the SDE miRNAs were subjected to a target-based pathway enrichment analysis to identify key nodes and edges in each set of data, and miRNA-mRNA regulatory networks were identified. We used the web-tool miRNet (13), which integrates data from eleven different miRNA databases and allow us to analyze miRNA-target interaction networks and functions.

HCV-Related Genes

We have analyzed the implication of the 21 SDE miRNAs in targeting host genes related to HCV infection, and nine out of 21 SDE miRNAs were identified. The most relevant miRNA in this analysis was has-miR-124-3p, which was upregulated in HCV-exposed individuals, and it targets 18 HCV infection-related genes. Here, we review the most relevant targets of this miRNA and their biological functions.

HCV interacts with components of the IFN alpha/beta pathway to inhibit the production of IFN and the establishment of an antiviral state (14). The hsa-miR-124-3p can target seven members of this signaling pathway: the toll like receptor 3 (TLR3), the inhibitor of nuclear factor kappa B kinase subunit epsilon (IKBKE), the eukaryotic translation initiation factor 2 alpha kinase 2 (EIF2AK2), the interferon-alpha/beta receptor 2 (IFNAR2), the tyrosine kinase 2 (TYK2), the protein inhibitor of activated STAT 1 (PIAS1), and RELA proto-oncogene (RELA). TLR3 is restricted endosomes, and it recognizes dsRNA associated with a viral infection to induce the activation of NF-kappaB and IFN production (15). Upon TLR3 engagement with its ligand, IKBKE is activated (15). The IKBKE inhibits T cell immune response (16), which is essential for regulating antiviral signaling pathways. Several viruses interact directly with and inhibit IKBKE/IKK-epsilon to prevent IRFs activation. IKBKE kinases activate several transcription factors such as the nuclear factor-kappaB (NF-kB). RELA is a subunit of the nuclear NF-Kb, which is involved in multiple aspects of innate and adaptive immune

functions (17). RELA is required during a key early phase after virus infection (18), where it is crucial for early IFN-beta expression and resistance to RNA virus replication. The coordinated action of NF-Kb together with other transcription factors leads to the induction of IFN and proinflammatory cytokines and to the establishment of the innate immune response (19). HCV has developed strategies to disrupt the induction of IFN and cytokine pathways through viral protein interaction, favoring viral propagation and presumably HCV chronic infection (19). Secreted IFNs bind and activate the IFNAR2 in an autocrine and paracrine manner, which leads to the induction of hundreds of interferon stimulated genes (ISGs). IFNAR2 gene has been identified as overexpressed in PBMCs of HCV chronic patients, and its expression significantly correlates with the effectiveness of interferon therapy independently of the viral load (20). TYK2 encodes a protein associated with the cytoplasmic domain of type I and type II cytokine receptors and IFNAR and promulgate cytokine and IFN signals by phosphorylating receptor subunits. As such, it may play a role in the anti-viral immunity. IFN activates the signal transducer and activator of transcription (STAT) pathway to regulate immune response. The protein inhibitor of activated STAT 1 (PIAS1) is a negative regulator of STAT1, being critical for the IFN-gamma- or beta-mediated innate immune response (21). PIAS1 has a dual function, acting at the virus-induced early singling stage and IFN stimulated amplifying stage. Thus, PIAS1 maintains proper amounts of type I IFNs and retains its magnitude when the antiviral response intensifies (22). EIF2AK2 is a serine/threonine protein kinase that play a key role in the innate immune response to viral infection as well as is involved in the regulation of signal transduction in different pathways as well as in the expression of cytokines and IFNs. It has an antiviral activity on HCV, among others.

On the other hand, through TLR3 activation, the PI3K/AKT signaling pathway can be induced to produce ISGs (23). HCV activates PI3K-Akt signaling to enhance entry, replication, and translation (24, 25). Three main factors of this pathway targeted by has-miR-124 are the phosphatidylinositol-4,5-bisphosphate 3-kinase catalytic subunit alpha (PIK3CA) and the AKT serine/threonine kinase 2 (AKT2) and 3 (AKT3). PIK3CA is the catalytic subunit of the PI3K, and it is involved in cell proliferation, apoptosis, and angiogenesis. Mutations within its gene have been identified in HCC in different populations (26). AKT2 and AKT3 are key effectors of the insulin signaling pathway and regulates cellular metabolism (27). HCV seems to activate AKT to apparently enhance the permissiveness of the cell for HCV infection (28). The assault by HCV on this pathway that regulates cellular growth and metabolism probably plays an important role in HCV pathogenesis.

HCV modulates the MAPK/ERK cascade for its own propagation (29). Eight genes within this pathway can be targeted by hsa-miR-124-3p: the growth factor receptor bound protein 2 (GRB2), SOS Ras/Rho guanine nucleotide exchange factor 1 (SOS1) and 2 (SOS2), A-Raf proto-oncogene, cytosolic serine/threonine kinase (ARAF), the GTPase neuroblastoma RAS proto-oncogene (NRAS) (30), the mitogen-activated protein kinase 11 (MAPK11), the protein phosphatase 2 scaffold subunit Abeta (PPP2R1B), and the cyclin dependent kinase inhibitor 1A (CDKN1A) also known as p21. This pathway can be active by receptor tyrosine kinases (RTK) such as the epidermal growth factor receptor (EGFR), which is a central controller of HCV viral entrance (31). The signal received by the receptor is integrated by the RAS/RAF/MEK/MAPK cascade reaction that activates the signal transduction pathway and activates transcription factors to regulate gene expression. GRB2 is the downstream effector of the receptor and, together with SOS1 and SOS2, regulates the action of RAS protein. Previous report has detected an interaction of the HCV NS5A protein with GRB2 to inhibit downstream mitogen signaling (32). In vitro assays of Huh7.5-infected cells (33) identified SOS1 as one internal driving factor leading to the extrahepatic manifestation of an HCV infection. SOS1 activate the MAPK signaling pathway through RAS activation. RAS proteins act as molecular switches and are involved in several signaling transduction pathways including MAPK and the previously mentioned PI3K. NRAS is one of the three RAS isoforms that control cell proliferation and growth. Once RAS is activated, it recruits and activates the protein kinases RAF, a family of enzymes (such as ARAF) that activates ERK signaling by phosphorylating MEK, which in turns activate MAPK. MAPK11 is also activated in the presence of an HCV core protein (34, 35). In vitro studies in human hepatoma cells have previously reported that a low level of Ras-ERK signaling activity is required for HCV RNA replication but that a complete inhibition is inhibitory (36). The protein

phosphatase 2 scaffold subunit Abeta (PPP2R1B) is a regulator of this pathway. Different viruses such as HCV interact with PP2R1B to specifically subvert key survival pathways for the host (37). Finally, CDKN1A is a regulator of the cell cycle and is frequently mutated in human cancers, such as HCC. In addition, the HCV core protein downregulates p21 in human hepatoma cells (38).

Therefore, the upregulation of hsa-miR-124 by an HCV infection will repress the RAS-MAPK signaling pathway at different levels to ultimately regulate HCV RNA replication.

An HCV infection requires multiple host signaling pathways to successfully infect the host cell. Our results suggest that the subverts of miRNAs are an additional strategy of HCV to maintain infection (39) as the upregulation of hsa-miR-124 in PBMCs by HCV downregulates IFN production at different stages.

References

1. Tuma P, Medrano J, Resino S, Vispo E, Madejon A, Sanchez-Piedra C, et al. Incidence of liver cirrhosis in HIV-infected patients with chronic hepatitis B or C in the era of highly active antiretroviral therapy. Antiviral therapy. 2010;15(6):881-6.
2. Thorpe LE, Ouellet LJ, Hershow R, Bailey SL, Williams IT, Williamson J, et al. Risk of hepatitis C virus infection among young adult injection drug users who share injection equipment. Am J Epidemiol. 2002;155(7):645-53.
3. Andrews S. FastQC—A Quality Control Tool for High Throughput Sequence Data. 2016.
4. Friedländer MR, Mackowiak SD, Li N, Chen W, Rajewsky N. miRDeep2 accurately identifies known and hundreds of novel microRNA genes in seven animal clades. Nucleic Acids Res. 2012;40(1):37-52.
5. Bonnet E, Wuyts J, Rouzé P, Van de Peer Y. Evidence that microRNA precursors, unlike other non-coding RNAs, have lower folding free energies than random sequences. Bioinformatics. 2004;20(17):2911-7.
6. Anders S, Huber W. Differential expression analysis for sequence count data. Genome Biol. 2010;11(10):R106.
7. Robinson MD, McCarthy DJ, Smyth GK. edgeR: a Bioconductor package for differential expression analysis of digital gene expression data. Bioinformatics. 2010;26(1):139-40.
8. Tarazona S, Furió-Tarí P, Turrà D, Pietro AD, Nueda MJ, Ferrer A, et al. Data quality aware analysis of differential expression in RNA-seq with NOISeq R/Bioc package. Nucleic Acids Res. 2015;43(21):e140.
9. Efron B, Tibshirani R. Empirical bayes methods and false discovery rates for microarrays. Genet Epidemiol. 2002;23(1):70-86.
10. Vlachos IS, Zagganas K, Paraskevopoulou MD, Georgakilas G, Karagkouni D, Vergoulis T, et al. DIANA-miRPath v3.0: deciphering microRNA function with experimental support. Nucleic Acids Res. 2015;43(W1):W460-6.
11. Karagkouni D, Paraskevopoulou MD, Chatzopoulos S, Vlachos IS, Tastsoglou S, Kanellos I, et al. DIANA-TarBase v8: a decade-long collection of experimentally supported miRNA-gene interactions. Nucleic Acids Res. 2018;46(D1):D239-D45.
12. Kanehisa M, Goto S. KEGG: kyoto encyclopedia of genes and genomes. Nucleic Acids Res. 2000;28(1):27-30.
13. Fan Y, Xia J. miRNet-Functional Analysis and Visual Exploration of miRNA-Target Interactions in a Network Context. Methods Mol Biol. 2018;1819:215-33.
14. Wang Y, Li J, Wang X, Ye L, Zhou Y, Thomas RM, et al. Hepatitis C virus impairs TLR3 signaling and inhibits IFN-λ 1 expression in human hepatoma cell line. Innate Immun. 2014;20(1):3-11.
15. Chau TL, Göktuna SI, Rammal A, Casanova T, Duong HQ, Gatot JS, et al. A role for APPL1 in TLR3/4-dependent TBK1 and IKKε activation in macrophages. J Immunol. 2015;194(8):3970-83.
16. Zhang J, Feng H, Zhao J, Feldman ER, Chen SY, Yuan W, et al. IκB Kinase ε Is an NFATc1 Kinase that Inhibits T Cell Immune Response. Cell Rep. 2016;16(2):405-18.
17. Liu T, Zhang L, Joo D, Sun SC. NF-κB signaling in inflammation. Signal Transduct Target Ther. 2017;2.
18. Wang J, Basagoudanavar SH, Wang X, Hopewell E, Albrecht R, García-Sastre A, et al. NF-kappa B RelA subunit is crucial for early IFN-beta expression and resistance to RNA virus replication. J Immunol. 2010;185(3):1720-9.
19. Meurs EF, Breiman A. The interferon inducing pathways and the hepatitis C virus. World J Gastroenterol. 2007;13(17):2446-54.

20. Liu N, Wen Y, Sun C, Liu P. Expression of IFNAR2 mRNA in peripheral blood mononuclear cells of patients with HCV infection. Acta Gastroenterol Belg. 2012;75(2):228-33.
21. Liu B, Mink S, Wong KA, Stein N, Getman C, Dempsey PW, et al. PIAS1 selectively inhibits interferon-inducible genes and is important in innate immunity. Nat Immunol. 2004;5(9):891-8.
22. Li R, Pan Y, Shi DD, Zhang Y, Zhang J. PIAS1 negatively modulates virus triggered type I IFN signaling by blocking the DNA binding activity of IRF3. Antiviral Res. 2013;100(2):546-54.
23. Chen K, Liu J, Cao X. Regulation of type I interferon signaling in immunity and inflammation: A comprehensive review. J Autoimmun. 2017;83:1-11.
24. Shi Q, Hoffman B, Liu Q. PI3K-Akt signaling pathway upregulates hepatitis C virus RNA translation through the activation of SREBPs. Virology. 2016;490:99-108.
25. Mannová P, Beretta L. Activation of the N-Ras-PI3K-Akt-mTOR pathway by hepatitis C virus: control of cell survival and viral replication. J Virol. 2005;79(14):8742-9.
26. Tornesello ML, Buonaguro L, Tatangelo F, Botti G, Izzo F, Buonaguro FM. Mutations in TP53, CTNNB1 and PIK3CA genes in hepatocellular carcinoma associated with hepatitis B and hepatitis C virus infections. Genomics. 2013;102(2):74-83.
27. Juntilla MM, Patil VD, Calamito M, Joshi RP, Birnbaum MJ, Koretzky GA. AKT1 and AKT2 maintain hematopoietic stem cell function by regulating reactive oxygen species. Blood. 2010;115(20):4030-8.
28. Liu Z, Tian Y, Machida K, Lai MM, Luo G, Foung SK, et al. Transient activation of the PI3K-AKT pathway by hepatitis C virus to enhance viral entry. J Biol Chem. 2012;287(50):41922-30.
29. Huynh VT, Lim YS, Tran SC, Pham TM, Nguyen LN, Hwang SB. Hepatitis C Virus Nonstructural 5A Protein Interacts with Abelson Interactor 1 and Modulates Epidermal Growth Factor-mediated MEK/ERK Signaling Pathway. J Biol Chem. 2016;291(43):22607-17.
30. Lang MF, Yang S, Zhao C, Sun G, Murai K, Wu X, et al. Genome-wide profiling identified a set of miRNAs that are differentially expressed in glioblastoma stem cells and normal neural stem cells. PLoS One. 2012;7(4):e36248.
31. Miao Z, Xie Z, Miao J, Ran J, Feng Y, Xia X. Regulated Entry of Hepatitis C Virus into Hepatocytes. Viruses. 2017;9(5).
32. He Y, Nakao H, Tan SL, Polyak SJ, Neddermann P, Vijaysri S, et al. Subversion of cell signaling pathways by hepatitis C virus nonstructural 5A protein via interaction with Grb2 and P85 phosphatidylinositol 3-kinase. J Virol. 2002;76(18):9207-17.
33. Wu ZY, Li JR, Huang MH, Cheng JJ, Li H, Chen JH, et al. Internal driving factors leading to extrahepatic manifestation of the hepatitis C virus infection. Int J Mol Med. 2017;40(6):1792-802.
34. Hayashi J, Aoki H, Kajino K, Moriyama M, Arakawa Y, Hino O. Hepatitis C virus core protein activates the MAPK/ERK cascade synergistically with tumor promoter TPA, but not with epidermal growth factor or transforming growth factor alpha. Hepatology. 2000;32(5):958-61.
35. Song X, Yao Z, Yang J, Zhang Z, Deng Y, Li M, et al. HCV core protein binds to gC1qR to induce A20 expression and inhibit cytokine production through MAPKs and NF-κB signaling pathways. Oncotarget. 2016;7(23):33796-808.
36. Gretton S, Hughes M, Harris M. Hepatitis C virus RNA replication is regulated by Ras-Erk signalling. J Gen Virol. 2010;91(Pt 3):671-80.
37. Guergnon J, Godet AN, Galioot A, Falanga PB, Colle JH, Cayla X, et al. PP2A targeting by viral proteins: a widespread biological strategy from DNA/RNA tumor viruses to HIV-1. Biochim Biophys Acta. 2011;1812(11):1498-507.
38. Shiu TY, Huang SM, Shih YL, Chu HC, Chang WK, Hsieh TY. Hepatitis C virus core protein down-regulates p21(Waf1/Cip1) and inhibits curcumin-induced apoptosis through microRNA-345 targeting in human hepatoma cells. PLoS One. 2013;8(4):e61089.
39. Li Q, Lowey B, Sodroski C, Krishnamurthy S, Alao H, Cha H, et al. Cellular microRNA networks regulate host dependency of hepatitis C virus infection. Nat Commun. 2017;8(1):1789.
